# Citizen science monitoring reveals links between honeybee health, pesticide exposure and seasonal availability of floral resources

**DOI:** 10.1038/s41598-022-18672-0

**Published:** 2022-08-22

**Authors:** Ben A. Woodcock, Anna E. Oliver, Lindsay K. Newbold, H. Soon Gweon, Daniel S. Read, Ujala Sayed, Joanna Savage, Jim Bacon, Emily Upcott, Katherine Howell, Katharine Turvey, David B. Roy, M. Gloria Pereira, Darren Sleep, Arran Greenop, Richard F. Pywell

**Affiliations:** 1 UK Centre for Ecology & Hydrology (UKCEH), Wallingford, Oxfordshire, OX10 8BB UK; 2 School of Biological Sciences, University of Reading, Reading, RG6 6UR UK; 3 UK Centre for Ecology & Hydrology (UKCEH), Lancaster Environment Centre, Library Ave., Bailrigg, Lancaster, LA1 4AP UK

**Keywords:** Agroecology, Biodiversity, Ecosystem services, Environmental impact

## Abstract

We use a national citizen science monitoring scheme to quantify how agricultural intensification affects honeybee diet breadth (number of plant species). To do this we used DNA metabarcoding to identify the plants present in 527 honey samples collected in 2019 across Great Britain. The species richness of forage plants was negatively correlated with arable cropping area, although this was only found early in the year when the abundance of flowering plants was more limited. Within intensively farmed areas, honeybee diets were dominated by *Brassica* crops (including oilseed rape). We demonstrate how the structure and complexity of honeybee foraging relationships with plants is negatively affected by the area of arable crops surrounding hives. Using information collected from the beekeepers on the incidence of an economically damaging bee disease (Deformed Wing Virus) we found that the occurrence of this disease increased where bees foraged in agricultural land where there was a high use of foliar insecticides. Understanding impacts of land use on resource availability is fundamental to assessing long-term viability of pollinator populations. These findings highlight the importance of supporting temporally timed resources as mitigation strategies to support wider pollinator population viability.

## Introduction

Insect pollinators are currently undergoing population declines linked to a number of factors including land use, agricultural intensification (including agrochemical usage), invasive species, diseases and climate change^1–5^. In the case of land use change, understanding its impact on resource utilisation by honeybee could provide insights into the long-term viability of pollinator populations. This would also provide an evidence base for developing mitigation strategies that can help address resource deficits for this key group delivering pollination services to crops and wild plants^1,4^. However, it has proved challenging to assess foraging plant resource utilisation by honeybees at these large scales limiting our understanding of how this could impacts upon population level processes (e.g. across a region the size of Great Britain).

For insect pollinators, in particular bees, the availability of foraging resources at landscape scales has a significant impact on population viability^6–9^. There are core calorific and nutritional requirements supported by pollen (to provide principally protein) and nectar (to provide carbohydrates) foraged upon by bees^10^. Limitations on either being likely to lead to population declines^11,12^. However, individual plants can vary considerably in nectar availability, as well as protein content and amino acid profiles^10,13,14^. This variability has the potential to affect key fitness metrics affecting pollinator survival^9,14,15^. Perhaps the most significant is how diet quality affects susceptibility to economically important parasites and diseases. In honeybees, diverse diets can increase their ability to resist diseases (i.e. immunocompetence)^8^, whilst the availability of high protein pollens originating from single plant species can improve tolerance to parasitic and viral infections^9,15^. The negative impact of poor diet on disease susceptibility is unlikely to act in isolation, being directly or indirectly impacted upon by a range of other environmental stressors. Of these, exposure to insecticides may pose a major risk in many agricultural systems where sub-lethal doses of pesticides weaken the immune system of honeybees increasing the likelihood of them becoming susceptible to diseases^16^. The negative effect of insecticides on bees can also be direct, with exposure resulting in immediate and long term toxicity effects^17,18^. However, while insecticides may be expected to have negative effects on bees other widely used agrochemicals considered to have low toxicity to honeybees^19^, such as fungicides and herbicides, may also have negative effects^20,21^. For example, while the very widely used herbicide glyphosate is considered to have low toxicity to bees from a regulatory perspective, a recent meta-analysis identifies its use with increased mortality of bees^21^. Similarly, the ubiquitously used azole fungicides (e.g. triazoles) can have unexpected synergistic interactions with other pesticides impacting negatively on bees^20,22^. Quantifying the extent to which these environmental drivers will impact on honeybees at national scales in real world agricultural systems has important implications for how we will manage these systems in the future.

Whilst diet quality may be of fundamental importance to bee health, land use affected by human activity is known to have negatively affected the availability of foraging resources on which they can feed^23^. This includes the loss of floristically rich semi-natural habitats from agricultural landscapes, as well as the role of herbicides and crop seed cleaning reducing the prevalence of flowering arable weeds^23–26^. This loss of general floral diversity may be compensated to some extent by the prevalence of mass flowering crops, including oilseed rape, that act as a highly abundant monofloral resource for many generalist pollinator species^3,27^. However, diets dominated by a few species of crop where landscape scale saturation occurs, may potentially lead to a dramatically simplified diet that have negative impacts on health^9,15,28^. Independent of this, the prevalence of agricultural land use has been linked with a reduction in the diversity of pollen foraged upon by honeybees^29^, as well as an associated reduction in the protein content of stored hive products vital to support larval development^30^. A characteristic of many semi-natural and agricultural landscapes are strong seasonal shifts in flower resource availability, leading to boom and bust periods for pollinators^31–34^. While pollinators forage dynamically across varying scales to compensate for such temporal resources variability, ultimately landscape diversity and quality places an upper limit on what can be extracted to support population growth^31^. In the case of honeybees, there is a greater requirement for pollen to support colony growth in the spring, while nectar becomes more important later in the year for maintaining colony size and for storage as honey^35^. As such, temporal variability in flowering resources may interact with key periods of specific nutritional requirements to have unexpected negative effects.

The quantification of bee diet through the assessment of pollen types returned to nests is a direct approach to understand patterns of resource availability and their impacts on population viability of insect pollinators. Until recently, applying such assessments at large scales has been impractical due to time and expertise limitations associated with microscopy based pollen taxonomy. With the advent of molecular approaches, large-scale quantification of pollen types returned to hives is now viable e.g.^36–40^. However, to apply this in a systematic way there is a need for model systems. Honeybees (*Apis mellifera*) are a practical model system, having an existing evidence base for their dependence on pollen diversity and quality^15,41^, being characterised by foraging ranges as large or larger than other bee species^32,42,43^, while also being a prominent model system in ecotoxicology^44^. Also, there exists a network of amateur and professional beekeepers who are willing to provide national scale, spatially and temporally referenced samples of honey from which pollen can be extracted and classified^39^.

**Figure 1:**
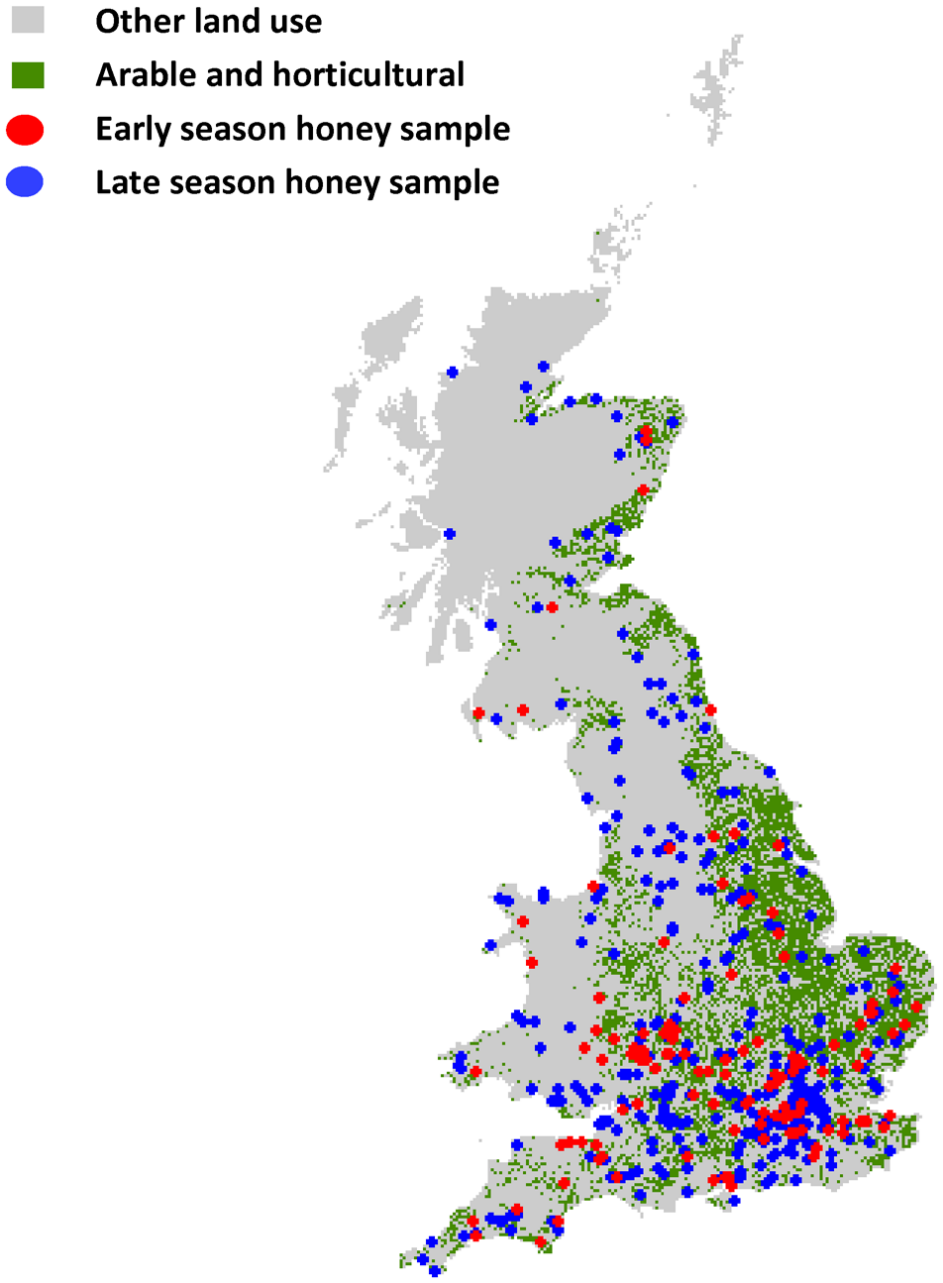
Location of the 527 early (collected in June and before: N = 119) and late (July and after:N = 408) honey samples collected and analysed to determine diet breath of honeybees in Great Britain. Map created in R: Version 3.6.3. (URL hhtp://cran.r-project.org).

In this study, we utilise 527 honey samples collected across Great Britain as part of the National Honey Monitoring Scheme (https://honey-monitoring.ac.uk/) (Fig. 1). This represents citizen science programs where members of the general public (specifically bee keepers) are vital to the collection of samples that underpin scientific research that would otherwise be impractical or too costly to achieve without their support. This science scheme represents one of many citizen science programs around the world that have engaged with bee keeper communities that often have a vested interest in understanding the ecology and threats posed to the hives and bees they manage^45–48^. A subset of the samples provided by these citizen scientist bee keepers were provided with meta-data that included information on prevalence of pests and disease, including *Varroa* mite infections within the hive. Environmental DNA (eDNA) from pollen suspended within the honey samples was identified using metabarcoding to quantify what the honeybees had foraged upon. Environmental DNA is collected without isolating a specific organism from an environmental sample, i.e. pollen suspended in honey assessed in aggregate rather than by separate species^49,50^. The environmental context of hive locations was assessed through spatial data on land use, cropping patterns and agrochemical applications. Using this data set we asked the following questions: (1) Does the prevalence of agricultural land use and its associated simplification of the floral community impact on the availability of forage plant species for honeybees and the complexity of their foraging patterns? (2) Does the prevalence of highly attractive sown forage crops, in particular *Brassica’s,* such as oilseed rape, correlate with lower honeybee diet breath? (3) Does a reduction in resource breath (i.e. the variety of forage plants) and agrochemical use correlate with colony susceptibility to the widespread and economically damaging infestations of the *Varroa* mite and Deformed Wing Virus. Here we use disease susceptibility as a metric for colony health.

## Results

### Foraging preferences of the honeybees

The majority of samples were from England (N = 467), with a relatively small number from Wales (N = 31) and Scotland (N = 34). *Brassica* crops (Brassicaceae), in particular oilseed rape (*B. napus*), turnip (*B. rapa*) and cabbage (*B. oleraceae*), have a close genomic relationship and their separation is unreliable^51^. Considered as an aggregate, the *Brassica* group was the dominant forage plants across the 527 samples (85.7%), followed closely by the common hedgerow species aggregate *Rubus* spp (Rosaceae; 80.2% of samples) as well as *Trifolium repens* (70.6%) which is ubiquitous in improved and other GB grasslands. The non-native flowering shrub/tree *Ligustrum ovalifolium* (Oleaceae)*,* which is widely grown in gardens, was the next most dominant forage plant found in 57.3% of honey samples. While these species were also the dominant forage plants in England, this was not true of Scotland and Wales where native species like *Filipendula ulmaria (Rosaceae)* and *Chamaenerion angustifolium* (Onagraceae; Scotland only) were more commonly foraged upon than *L. ovalifolium. For both Scotland and Wales* the aggressively invasive species *Impatiens glandulifera* (Balsaminaceae) was also within the top 5 most commonly foraged upon plants, found in respectively 58.8 and 67.7% of samples.

#### Diet breath of the honeybees

**Figure 2:**
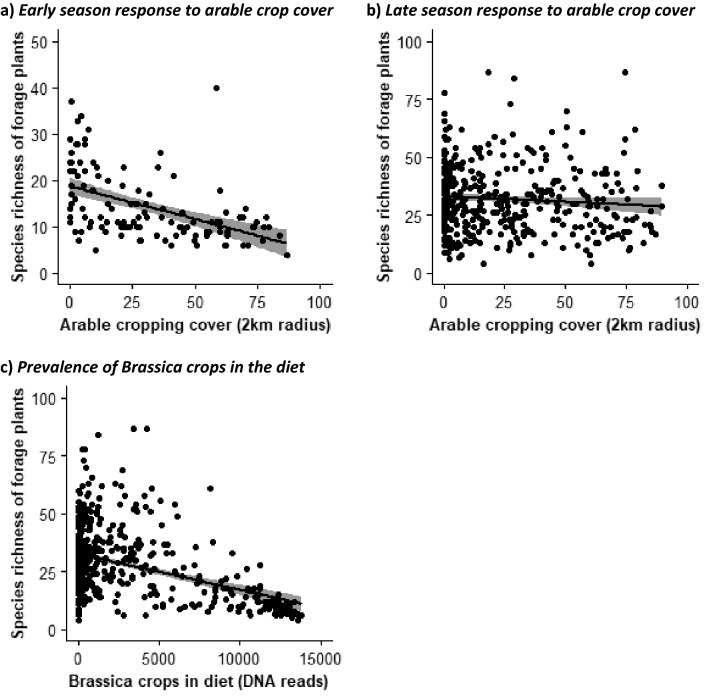
Effect of surrounding land use on the diet breadth (species richness of forage plants) of honeybees in Great Britain. Response of diet breadth to arable and horticultural crop cover are shown for the (**a**) early (June and before) and (**b**) late (July and after) parts of the year, as well as (**c**) to the prevalence of *Brassica* crops (including Oilseed rape) found in honeybee diets.

The species richness of plants foraged upon by honeybees from unique hives was significantly affected by an interaction between surrounding arable crop cover and the season in which hives were collected (F_1,520_ = 4.28, *p* = 0.04). The breadth of plants foraged upon to produce honey during the early season (≤ June) was typically lower than that seen latter on in the year (≤ July) (Fig. 2a and b). There was also far more variability between hives during the late season. However, even with this lower species richness, the breadth of the diet of honeybees during the early season negatively correlated with overall arable cover surrounding hives (Fig. 2a). For late season honey, the strength of this correlation was close to zero (Fig. 2b). The arable cropping rotations within a 2 km radius of hives (F_1,520_ = 7.17, *p* = 0.001), defined using a PCA axis of non-insect attractive crops, was negatively correlated with the diet breadth of honeybees. This pattern suggested that crop rotations domination by winter wheat were associated with the lowest diet breadth of foraging plants (Fig. S2a; supplementary information Table S1). There was evidence for a contraction in diet breadth where honeybee diets became increasingly dominated by *Brassica* crops (including oilseed rape). Here there was a strong negative correlation with the species richness of other forage plants within the diet of the bees and the summed DNA read counts of these crops derived from analysis of the honey samples (F_1,520_ = 41.4, *p* < 0.001; Fig. 2c). Finally, there was a negative correlation with flowering habitat and land use (PCA axis 2) (F_1,520_ = 21.8, *p* < 0.001; Fig. S2b). This relationship was based on PCA axis scores that suggested that landscapes dominated by improved grasslands were more likely to be characterised by a reduced species richness of foraging plants compared to those dominated by urban land use (supplementary information Table S2). There were no other significant interactions, nor was there evidence of spatial autocorrelation in model residuals (Morran’s I = − 0.053, Exp. = − 0.002, Var. = 0.003, *p* > 0.05).

#### Between hive complexity of trophic interactions

**Figure 3:**
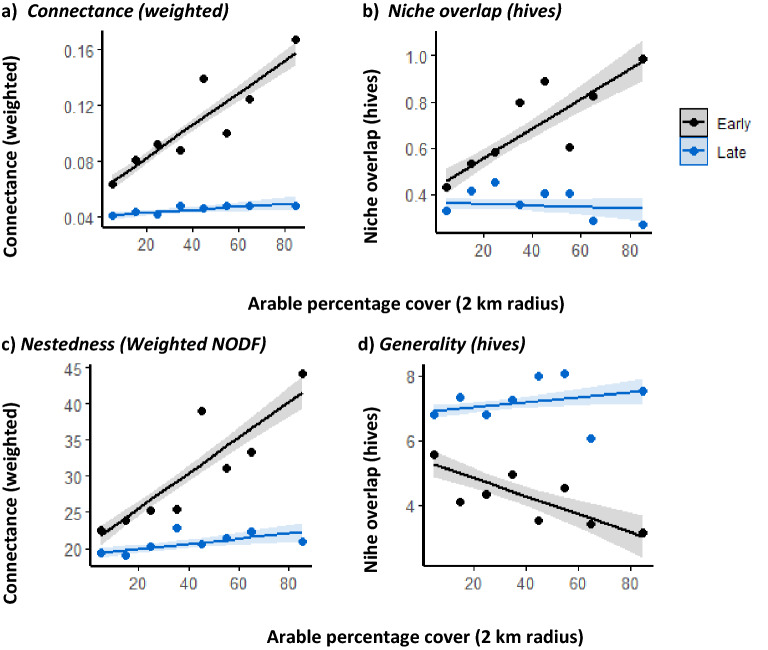
We derived bipartite food webs that describe the foraging associations between individual hives and plants as determined from DNA barcoding of pollen found in honey. These webs were derived depending on the time honey was harvested (early ≤ June or late ≥ July) and the extent to which the landscape was dominated by arable and horticultural land cover. We used these bipartite foraging webs to derive metrics describing the structure of these feeding relationships. These metrics were weighted connectance (realised proportion of possible links between hives and plants), weighted NODF nestedness (the tendency for hives to forage on subsets of plants utilised by better-connected hives, where larger values indicate increased nestedness), niche overlap (mean similarity of interaction patterns for hives with plants) and generality (mean effective number of plants foraged upon per hive). These graphs show how these metrics change in response to the cover of arable and horticultural land in the landscape.

**Figure 4:**
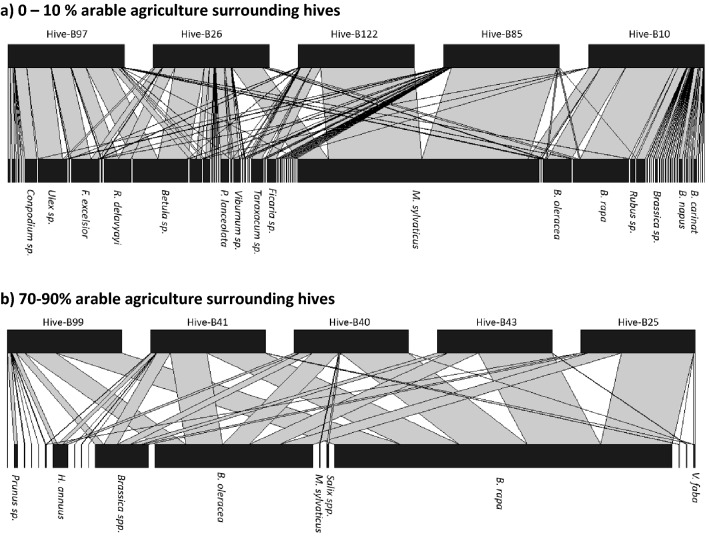
We derived bipartite food webs determined from DNA barcoding of pollen from honey. This allowed us to establish foraging associations between hives and plants in landscapes with different extents of arable and horticultural land. Here we show an examples of these webs of foraging relationships for five randomly picked hives (top rectangles) and forage plant (bottom rectangles) located in landscapes of either low (0–10% ) or high (70–90%) arable and horticultural cover. Both webs are determined from honey samples collected early in the season (June or before). We have standardised these webs to show only five randomly chosen hives as different landscapes had different numbers of hives which would make comparing an overall interaction web hard to interpret. Only plant names representing important feeding relationships are included, where: B. = *Brassica*, H. = *Helianthus*, M. = *Myosotis*, V. = *Vicia*, F. = *Frazinus*, R. = *Rhododendron*, P. = *Plantago*.

Following the derivation of bipartite foraging relationships between hives and flowering plant species, we found that connectance (F_1,12_ = 40.9, *p* < 0.001, Figs. 3a and 4), nestedness (F_1,12_ = 24.3, *p* < 0.001, Figs. 3b and 4), niche overlap (F_1,12_ = 15.9, *p* = 0.002, Figs.3c and 4), and generality (F_1,12_ = 8.11, *p* = 0.01, Figs. 3d and 4) all responded to an interaction between arable crop cover and season. For connectance, nestedness and niche overlap this relationship was characterised by strongly positive correlations early in the season, while the later season was, in all cases, characterised by a correlation close to zero (or slightly negative in the case of niche overlap). In contrast, the generality of the hives feeding relationships declined with increasing arable crop cover early in the season, but again was close to zero in the late season.

#### Disease infection rates

**Figure 5:**
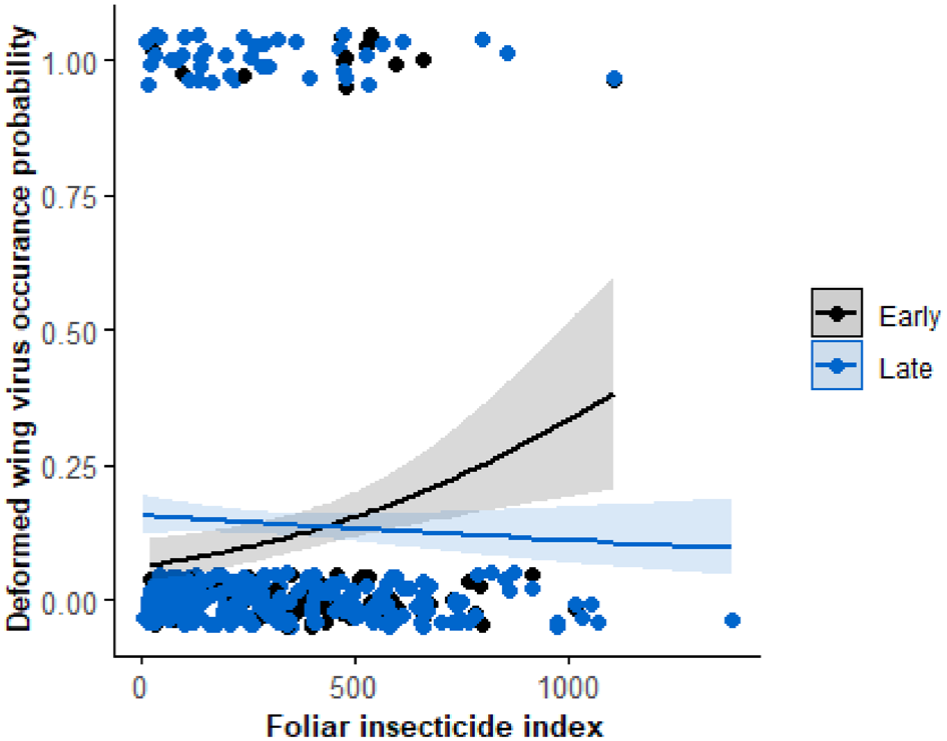
In this figure we show how foliar applied insecticide use surrounding hives is correlated with the occurrence of the deformed wing virus. For honey collected early in the year (June and before—black circles) there is a positive correlation with the foliar insecticide index. This corresponds to the period where agrochemicals are most widely used on crops. However, this pattern disappears for late season honey samples (collected July and after—blue circles) collected when agrochemical use is relatively low. The trend lines showing these correlations include uncertainty in the prediction (confidence intervals) as a grey area.

The probability of hives having *Varroa* infestations at the time of honey harvesting was not found to respond to diet quality in terms of the species richness of plants foraged upon, nor was it affected by the strength of agrochemical exposure (*p* > 0.05). Agrochemical exposure was defined by the direct effect foliar insecticide (foliar insecticide index), as well as the average application rates within 2 km of hives of the potentially synergistic Triazole fungicides or the herbicide glyphosate (*p* > 0.05). No interaction terms were found to be significant. The probability of symptomatic expression of the DWV was significantly correlated with an interaction between the foliar insecticide index and season (F_1,373_ = 3.73, = 0.05; Fig. 5), but not diet breadth. For hives where honey was collected early in the year, there was an increased probability of the symptomatic expression of the virus where the foliar insecticide index of the surrounding landscape increased. This relationship was absent in the latter season. There were no other significant single or interactions effects found to significantly affect the expression of this virus, including those for predicted average Triazole and glyphosate applications. There was no spatial autocorrelation in the model (Morran’s I = 0.049, Exp. = − 0.003, Var. = 0.001, *p* > 0.05).

## Discussion

In this study, we have demonstrated how beekeeper citizen scientists combined with lab based metabarcoding analysis of pollen DNA can provide insights into the factors affecting the viability of honeybees and their associated crop pollination service. While honeybees may potentially compete with wild pollinators^52–54^, they have sufficient fundamental similarities to act as a model system for inferring general impacts of land use intensification on pollinators. Ultimately, they provide access to systematically collected large-scale foraging data at a scale normally outside of the scope of most research programs. We have shown how agricultural land use and management are factors affecting honeybee diets, the resilience of their inter-hive feeding relationships and even impacting susceptibility to disease.

### Agricultural land use impacts on forage plant utilisation

In agreement with Alburaki et al.^29^, the extent of arable agricultural land use surrounding hives had a negative impact on the diet breath of the honeybees in terms of the range of plant species they fed upon during the early part of the year. This effect was slightly more pronounced where cropping rotations were dominated by winter wheat, one of the most widely grown and intensively managed of the arable crops. This negative effect of arable cropping land use was seen in terms of it being not only a predictor of the total number of forage plants, but also as a determinant of complex characteristics of resource overlap and utilisation between competing hives. As the cover of arable cropping increased, hives typically foraged on a greater proportion of the plants utilised by better-connected hives (Nestedness), while the mean effective number of plants foraged upon by individual hives simultaneously decreased (Generality). The similarity of interaction patterns with the types of plants on which different hives foraged also increased with arable cover (niche overlap). These effects are characteristic of a general simplification of resource choices within arable dominated landscapes. This effect has been reported in the US where there is increased patterns of foraging overlap for the most abundant plants within the diet of honeybees^55^. A likely effect of this is that hives may have been more likely to utilise what limited plant species were present as arable cropping came to dominate landscapes. This impact of arable agriculture on both foraging opportunities with increased arable cover reflects the historic loss from these production systems of flowering plants^14,24^. It is likely that this has affected not just diet breath by limiting the available number of species to forage upon, but also resource quality in terms of nectar availability and protein content^14,30^.

While the impact of arable crop cover was pronounced, its effects were only found during the early part of the season, during June or before. This strong seasonal variation in resource utilisation by honeybees has previously been identified^31,56^. As the season progresses, the availability of plants in flower generally increased in North Western European landscapes and any negative effect of arable land use on measures of hive diet breath or the complexity of foraging relationships may tend to disappear. Although hedgerow plants and some trees are important foraging resources early on in the year, early season arable systems are limited in their availability of flowering plants in general^14,33^. In contrast, plants flowering in July and August provide some 60% of nectar production available for bees to forage upon in British landscapes^14^. One driver of this low availability of flowering plants in arable dominated landscapes has been the impact of intensive farm management practices on the persistence of annual weed species^14,23,25,26^. Such weed species in arable ecosystems are potentially important foraging resources, especially when mass flowering crops are not in flower^34^. The loss of these often-early flowering species has led to important ‘drought’ periods of floral resource deficiency in these systems. Such early season deficits in resource availability may be a particular threat to honeybees, and potentially other eusocial species, by limiting resource availability as a point in the season where colony growth should be maximised^35^. Of these other land uses, there was some suggestion that urban environments may be likely to provide a greater diversity of potential plant species to forage upon, something reported in several other studies^29,57^.

### Contraction in diet breath and mass flowering crops

Mass flowering crops, in particular oilseed rape, represents an important foraging resource for many insect pollinators within GB agricultural landscapes^3,27^. In the case of honeybees, oilseed rape pollen may be particularly rich in essential amino acids compared to other mass flowering crops like field beans, including leucine, valine, and isoleucine^13^. This nutritional profile of both nectar and amino acids has been seen to drive preferential selection by honeybees^13^. At least for honeybees, the loss of oilseed rape can reduce survival. Di Pasquale et al.^58^ suggests that pollen diversity is less important than the loss of such species that individually have a high nutritional value as well as being extremely abundant. We found that as *Brassica* crops become a more significant part of the diet of honeybees their overall diet range in terms of what other plants they forage upon contracted. It therefore seems likely that in the presence of mass flowering crops, such as oilseed rape, may cause bees to neglect other foraging resources. Such preferential selection by pollinators could reduce diet breath and may have indirect and unexpected impact on bee health. Diets lacking pollens from a range of different plants may have synergistic effects on bee health to such an extent that their loss may destabilize these communities^8,15,59^. In addition, this may have consequences for populations of wild flowering plants by reducing opportunities for pollination events as bees are drawn to high resource value fields of such mass flowering crops^60^.

### Resource breadth, agrochemicals use and the incidence of pests and disease

There is a significant evidence to suggest polyfloral diets play an important role in supporting bee health^8,15,59^. However, we found no evidence that diet breadth affected either *Varroa* mite infestation rates or the symptomatic expression of DWV, the commonest virus reported across the data set. It is likely that there is complexity underlying the role of polyfloral diets, with only a sub-set of plants making a significant contribution to maintaining bee health. This may be through either their improved nutrient profiles or because of direct disease inhibiting effects^15,61^. However, at least in the latter case, toxic chemical defences of plants may be as likely to have negative consequences for plant-honeybee interactions^62^. Although hives with broad diets would (through a sampling effect) be more likely to include beneficial species^63^, it is probable that the relative contribution to the diet of these species is an important determinant of their value^61^.

We also assessed the impact of agrochemical stressors on symptomatic expression of these two diseases, focusing not only on direct effects associated with foliar insecticides^16^, but synergistic consequences of exposure to other agrochemical classes^21,64,65^. While beekeepers do apply acaricides as a means of control rather than complete eradication of *Varroa* mite infestations (e.g. pyrethroids like flumethrin), the widespread resistance of *Varroa* to these products has meant a shift towards control using acids (e.g. oxalic or formic acid) and natural oils^66^. We found no evidence that *Varroa* infestation rates were affected by exposure risk to any of the considered agrochemicals. However, the symptomatic expression of DWV was positively correlated with the foliar insecticide index during the early part of the year. The identification of this effect in June and before is likely linked to this being the main period of insecticide use on crops, as well as when hives may by more vulnerable to pesticide exposure following the over wintering period. The asymptomatic presence of DWV is thought to be present in many hives, although its symptomatic expression may be considerably increased though synergistic interactions with *Varroa* infestations^16,67^. There was no evidence of synergistic interactions between the insecticide use index and the Triazole fungicides or glyphosate herbicide use. The absence of such effects runs counter to a recent meta-analysis of synergisms between agrochemicals^65^ as well as focused work on these plant protection products in particular^21,64^. It is possible that our use of proxies to define agrochemical exposure based on application rates surrounding hives^68^ may lack the sensitivity to identify such synergisms. Indeed, future work focusing on the direct testing of residues from stored hive products may provide a more effective approach to quantifying agrochemical exposure risk^69^.

## Conclusions

 Honeybees represent a useful model species to indicate the stressors impacting on insect pollinators more widely, in particular central place foragers such as bumblebees and solitary bees. Indeed, given the typically large foraging ranges of honeybees compared to wild species, it is likely that the observed trends here may well be extrapolated for smaller solitary bees which more typically forage less than 1 km from nests^32,70^. This study has highlighted that there may be a restriction in the availability of floral resources available to honeybees in the early season, and by extension pollinators in general, in landscapes dominated by arable agriculture. This effect was not found in the later part of the year. The early season encompasses a period during which perennial species, often established though agri-environmental schemes to provide foraging resources for bees, are yet to reach their peak flowering^24,33^. The impact of limited resource availability within intensively farmed landscapes may also be counteracted by the occurrence of large blocks of early mass flowering crops, such as oilseed rape. While often considered an important resource for bees in agricultural systems^3,27^, their prevalence in diets may act to further contract diet breadths for species already foraging in a resource-limited landscape. A risk associated with oilseed rape is that while it may be an important source of pollen and nectar in the early season, it is one of the most intensively managed of the GB crops in terms of pesticide use and so may representing a potential exposure risk to bees^71^. While there was no direct evidence that diet breadth affected bee health, other work has highlighted its importance^8,15,59^. This study emphasises the need for more careful consideration of management practices to mitigate the impacts of arable crop management on pollinators. In particular, there is a need to identify where temporal mismatch in resource availability occurs and develop new agri-environmental seed mixes that can overcome these deficits. While early flowering annuals may be harder to manage for any famers, needing annual re-establishment, this may be a vital part of this solution for maintaining pollinator populations.

## Methods

In 2019 beekeepers across Great Britain provided 527 honey samples (Fig. 1) as part of a citizen science initiative, the National Honey Monitoring Scheme (https://honey-monitoring.ac.uk/). Samples were collected by directly scraping honey from recently filled storage cells on the edges of recently filled combs within hives. These honey samples were then returned to the scheme with associated spatial and temporal meta-data. A smaller subset of these honey samples (N = 377) included additional meta-data on the colony health, such as the presence of *Varroa* mite infestation (Fig. S1). While *Varroa* is found in most colonies, the symptomatic expression of viral infections is far more variable making it a useful indicator for understanding disease susceptibility in response to variation in environmental drivers. Although symptomatic expression for a range of viruses was recoded, only Deformed Wing Virus (DWV) was reported with sufficient frequency to be considered in subsequent analyses (52 of 377 samples). Honey samples were split into two temporal batches: early season (Up to June; N = 119) and late season (from July onwards; N = 408). This broadly corresponds with the two main harvests of honey typically produced by apiaries and relates to the seasonal variation in resource availability common to GB agricultural systems^24,33^. In particular there can be a lack of flowering plants early in the year, although this gap may be filled by oilseed rape production in some agricultural landscapes^34^.

### Forage plant identification by DNA barcoding of pollen in honey

Although honey is derived from nectar, each sample is contaminated with pollen grains derived from the plants that bees foraged upon, as well as trace quantities of other environmental DNA from plant nectar, bacteria and fungi. The pollen grains were extracted allowing subsequent DNA metabarcoding and identification of plant species using an established informatics pipeline described in detail in Oliver et al.^39^. In summary, pollen was concentrated from honey using a vacuum filtration system (Nalgene) utilising 47 mm mixed cellulose esters membranes (pore size 1.2 µm). Total DNA was extracted using the DNeasy PowerPlant Pro Kit (Qiagen, Hilden, Germany), with an additional proteinase K step alongside homogenisation to aid complete lysis. Resultant DNA was quantified using a Nanodrop One spectrophotometer (Thermo Scientific, Waltham, MA, USA) and ~ 10–20 ng template used in a PCR reaction to amplify the Internal transcribed spacer region 2 (ITS2) of plant nuclear ribosomal DNA. Finally, normalisation and sequencing of amplicons was undertaken through the Illumina MiSeq platform with the MiSeq Reagent Kit v3 (Illumina Inc., San Diego, CA, USA). Raw sequence data were processed using the HONEYPI bioinformatics pipeline as described in Oliver et al.^39^. Taxonomically comparable amplicon unit sequence variants (ASVs) were phylotyped following species level rarefaction within the phyloseq package within R 3.6.3^72^. From this a data matrix describing honey sample by abundance of DNA reads for each plant species was produced. The summed DNA reads of Brassica crops, a category which included oilseed rape as the main UK mass flowering crop, was derived as a covariate for subsequent analyses.

### Landscape structure

Honeybees forage over potentially large distances, although on average feed within 2 km of hives^32,42,43^. To provide an indication of foraging resource surrounding each hives to this distance, we derived a range of measures of surrounding land use. We used the 2019 UKCEH Land cover map at a 20 m raster pixel resolution combined with the UKCEH Land Cover plus Crops map^73^ to derive three metrics of land use. The first was arable and horticultural percentage cover (hereafter called arable cover). This represents a fundamental descriptor of land use in the UK. The second was a principal components analysis describing the first two axis of variation determined from the cover of non-insect attractive crops, specifically maize, wheat (winter and spring separately), barley (winter and spring separately), sugar beet, potatoes and other crops (a summed category of crops not defined by the previous). Note this excluded the flowering crops of oilseed rape and field beans. This explains variation beyond simply total arable crop cover, while acknowledging underlying correlations between these cropping patterns that make their use as individual covariates in subsequent models impractical. Thirdly, we derived the first two axis of variation of a principal components analysis based on the land use cover of habitats likely to be important for foraging bees^14^. These were the cover of mass flowering crops (oilseed rape and field beans), woodlands, flower rich habitats (including heather, heather-grass as well as unimproved calcareous and neutral grassland), improved grassland (receiving inorganic fertiliser) and (sub-) urban land use. Based on an assessment of variable inflation factors in subsequent analyses, the first two axis of variation of both of these PCAs (crop and land use) were characterised by strong multi-colinearity with arable cover VIF > 8.0;^74^ and so were excluded from subsequent analyses.

### Agrochemical exposure risk

The intensity of agrochemical use was defined using the UKCEH Land Cover plus: Pesticides 2012–2017 map^68^. The mean active ingredient weight per hectare of foliar spray insecticides (alphacypermethrin, deltamethrin, chlorpyrifos, cypermethrin, dimethoate, esfenvalerate, lambdacyhalothrin, pirimicarb, taufluvalinate, zetacypermethrin, fosthiazate, oxamyl, pymetrozine) were determined. This map is based on average applications over a 5-year rotation period and was defined within a 2 km radius of each hive. While ideally pesticide exposure for the 527 honey samples would have been directly assessed by residue analysis this was prohibitively expensive, however a sub-set of 100 samples was assessed for agrochemical residues from 2019. While this represents a limited sub-set of active-ingredients, the probability of detecting these in honey was largely predicted by the application rates around hives based on the UKCEH Land Cover plus: Pesticides 2012–2017 map (Supporting Information Table S7).

UKCEH Land Cover plus: Pesticides 2012–2017 map we derived a Foliar Insecticide Impact index (FII) that represents a composite estimate of the impact of foliar sprayed non-systemic insecticides based on the Environmental Impact Quotient (EIQ)^75^. This index has previously been used to assess the impacts of insecticides on bees^3,17^. The FII was defined as:

1 $$ FII = \mathop \sum \limits_{i = 1}^{ai} \left( {Z_{ai} \times P_{ai} } \right) \times \left( {\frac{{M_{ai, R} }}{{A_{R} }}} \right) $$

where: Following Kovach et al.^75^ *Z*_*ai*_ is the toxicity of the active ingredient (*ai*) to bees where each active ingredient is scored as high (score of 5 where oral LD_50_ < 1 µg bee^−1^), medium (score of 3 where 100 µg bee^−1^ > oral LD_50_ > 1 µg bee^−1^) or low (score of 1 where oral LD_50_ > 100 ug bee^−1^). This ratio of 5:3:1 is part of the EIQ. *P*_*ai*_ is the active ingredient plant surface half-life estimated to be a quarter of the soil deterioration half-life (DT_50_)^76^. All values used were derived from the Pesticide Properties Data Base^19^. *M*_*ai, R*_ is the mass of active ingredient applied with the 2 km radius surrounding hives (region *R*), and *A*_*R*_ is the area within that same 2 km radius. Note, while neonicotinoid seed treatments are widely thought to have a negative impact on bee populations they were not in use in arable cropping systems in the UK in 2019^3^.

Although insecticide use represents the immediate risk to honeybees, other agrochemicals of relatively low toxicity may have unexpected interactions that can alter the sensitivity of bees to insecticides^65^. To account for this we have focused on compounds that have been identified in the literature as being associated with either synergistic negative impacts azole fungicides—e.g.^20,77,78^ or unexpectedly high real world toxicity to bees not predicted in the regulatory process e.g. glyphosate—^21^). These two groups of non-insecticide pesticides are ubiquitously used in the UK farming environment, with glyphosate being the dominate herbicide used on the principal mass flowering crop oilseed rape grown in the UK (63.2% respectively by weight of active ingredient), while azole fungicides comprise 82.3% by weight of the fungicides used on this same crop. Both are also widely used on wheat crops, although as non-flowering crops this are not as attractive to bees and so exposure risk is likely lower^79^. To account for this we also derived the mean application rates of the widely used herbicide glyphosate, as well as the combined application rate of triazole fungicides, both of which have been identified as having interactive effects. Both of these have been linked to potential negative effects on honeybees as part of interactions with insecticides^21,64^.

### The complexity of foraging associations

We derived bipartite food webs based on the foraging associations between individual hives and plants based on the DNA barcoding of pollen from honey. These were derived for randomised sub-sets of hives picked from 14 sub-groups of the overall dataset of 527 hives. These sub-groups were based on hives where honey was collected early (≤ June) or late (≥ July) season within the following band classes of 0–10%, 10–20%, 20–30%, 30–40%, 40–50%, 50–60%, 60–70% and 70–90% arable cover. Again, we focus on arable cover as the major axis of land use variation within GB. The number of hives in each of these 14 sub-groups varied (Supporting Information Table S3). To account for this we undertook 100 random selections of five hives from each of these sub-groups to produce bipartite foraging webs. Using the Bipartite package in R 6.3^80^, we then derived average values of a sub-set of metrics describing bipartite web structure and averaged these across these 100 random picks. These metrics were weighted connectance (realised proportion of possible links between hives and plants), weighted NODF nestedness (the tendency for hives to forage on subsets of plants utilised by better-connected hives, where larger values indicate increased nestedness), niche overlap (mean similarity of interaction patterns for hives with plants) and generality (mean effective number of plants foraged upon per hive) (Supplementary Information Table S5). In all cases, they provide key insights into resource utilisation and specialisation. Metrics were weighted by the number of DNA read counts.

### Statistics

We assessed the response of the diet breath (species richness of plants foraged upon by the honeybees determined by DNA barcoding) using generalised least squares regression in response to the covariates: season (categorical early or late), summed arable cover, cropping land use (PCA axis 2), habitat land use (PCA axis 2), *Brassica* pollen in diet and the foliar insecticide index (FII) (Supporting Information Table S4). All pairwise interactions, as well as triplicate interactions of the latter covariates with season were included. The model was fitted within the nlme package in R 3.6.3 with a spatial correlation structure defined with the ‘corSpatial’ function using X and Y spatial coordinates of hive locations^81,82^.We accounted for within-group heteroscedasticity with a weighting of 1|Season. A Box-Cox transformation of species richness was used to normalise the response. All models were simplified using deletion of least significant effects and standard model checks to ensure model assumptions were not violated. This included a formal test for spatial autocorrelation of residuals using Morran’s I as well as inspection of a correlelogram.

The probability of hives suffering *Varroa* infestation and DWV infection (both as separate binomial responses) were assessed in response to: (i) season (early or late); (ii) diet quality, defined by the richness of plants foraged upon (Ln N + 1); (iii) the foliar insecticide index based on a 2 km radius surrounding the hives; (iv) the summed application of Triazole fungicides; (v) summed application of glyphosate (supporting information Table S5). As before, all pairwise interactions were assessed, as well as triplicate interactions with season. This analysis was performed using a general linear model with binomial error and logit link. Model simplification and model checks were as before. As no evidence of spatial auto-correlation was detected, no further correction was required in the model specification to account for this.

Finally, each of the four metrics of bipartite web structure (connectance, NODF nestedness, niche overlap and generality) were tested using a linear model against the covariates of season and arable cover, as well as their pairwise interaction (supporting Information Table S6). Arable cover was defined as the mid-point of the land use categories from which the random bipartite web picks had been drawn (5, 15, 25, 35, 45, 55, 65 and 80%). As each response was an average of multiple randomisations from hives drawn across the country accounting for spatial autocorrelation was not used. In all analyses we weighted the response based on the number of hives in each land use category for the early or late season from which the original randomised hive draws came from. Model simplification and checks followed the same approaches as above.

## Supplementary Information

Supplementary Information.

## Data Availability

All data used in the analyses are included within the supporting information tables S4, S5 and S6. A summary of the DNA metabarcoding species information is given at https://doi.org/10.5285/e9ec63be-3f2b-4d1b-b9bf-77ca2b96c7f5.
