# Supplementary material for: Citizen science monitoring reveals links between honeybee health, pesticide exposure and seasonal availability of floral resources
Source: Sci Rep. 2022 Aug 22;12:14331. doi: 10.1038/s41598-022-18672-0 (PMC9395358; doi:10.1038/s41598-022-18672-0)
Supplement: Supplementary file 1 — Supplementary Information. [file 41598_2022_18672_MOESM1_ESM.pdf]

Supplementary information

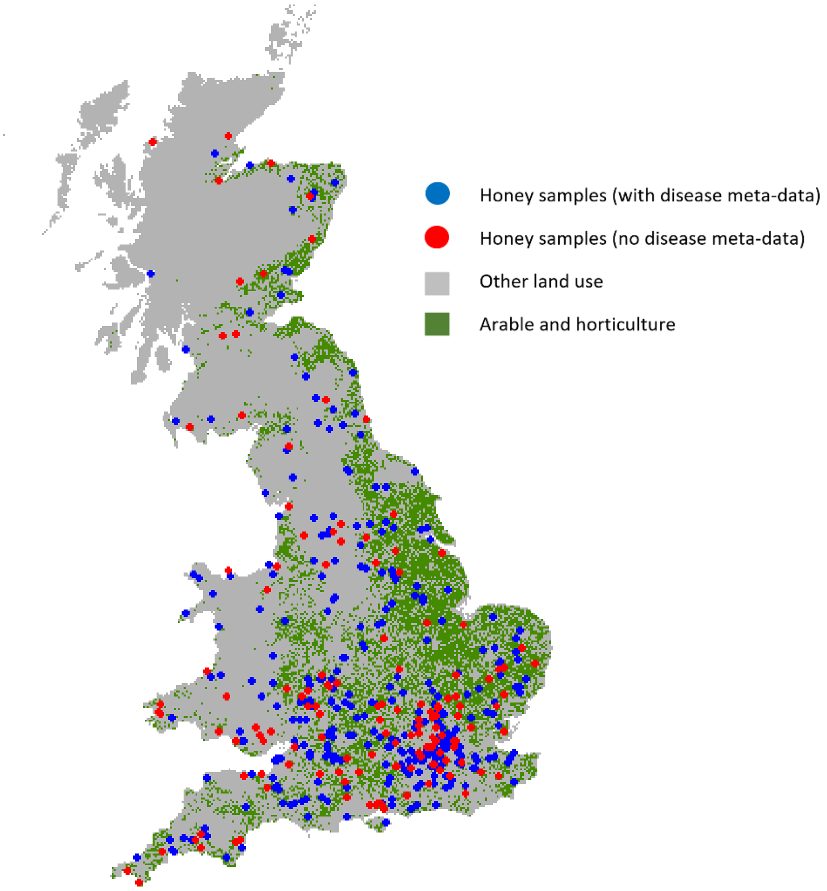

Fig. S1. Location of the sub set of 377 honey sampels where meta-data detailing incidence of symtomatic disease expression was provided (black circles) and for those samples where it was absent (red circles). Of these the most frequently reported were infestatins of the *Varroa* mite and the defromed wing virus. Map created in R: Version 3.6.3. (URL hhtp://cran.r-project.org).

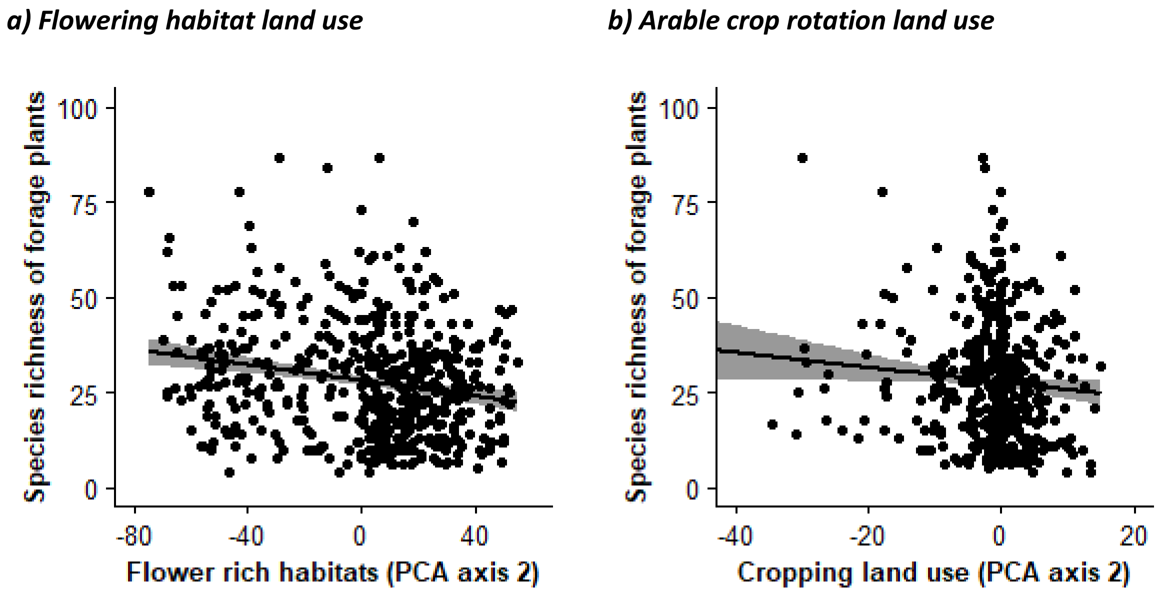

Fig S2. Effect of surrounding land use on the diet breath (species richness of forage plants) utilised by honeybee hives in Great Britain.

Table S1. PCA axis scores for the assessment land use change based on the percentage cover of non-nectar producing arable crops present within 2 km of hives. Only the second PCA axis scores were included as covariates in subsequent analyses due to evidence of intercorrelations with overall arable cropping cover.

	PC1 axis score	PC2 axis score
Maize	-0.12	-0.04
Winter Wheat	-0.9	0.37
Winter Barley	-0.23	-0.17
Spring Wheat	-0.07	-0.05
Spring Barley	-0.27	-0.84
Other crops	-0.19	-0.35
Sugar Beet	-0.05	0.02
Potatoes	-0.04	-0.04

Table S2. PCA axis scores for the assessment land use change based on habitats likely to be important for foraging honeybees based on the occurrence of flowering plants within 2 km of hives. Only the second PCA axis scores were included as covariates in subsequent analyses due to evidence of intercorrelations with overall arable cropping cover.

	PC1 axis score	PC2 axis score
Mass flowering crops	-0.08	0.02
Urban / suburban	-0.61	-0.79
Woodland	-0.23	0.14
Flower rich semi-natural	-0.03	0.02
Improved grassland	-0.75	0.59

Table S3. The number of hives from which honey samples were provided in each of the arable land cover classes used to derive bipartite foraging webs. In all cases repeated random picks of subsets of 5 hives were used to produce average metrics of connectance, nestedness, niche overlap and generality.

**Arable crop cover**	**Early season**	**Late season**
0-10 %	38	175
10-20 %	12	47
20-30 %	18	47
30-40%	9	33
40-50 %	11	26
50-60 %	12	26
60-70 %	9	24
70-90 %	10	30

Table S4. This table provides raw data used in the main analysis for the response of floral species richness of pollen found within the honey samples. See methods section for description of derivation of these covariates. Spatial coordinates (X and Y) have been de-resolved for the purposes of removing the locations of individual beekeepers.

Species richness of pollen in Honey (SR)	SR BoxCox	Season	Arable cover (2km radii)	Non flowering crop PCA2	Flowering habitat PCA2	Brassica crop DNA reads	FII insecticide index	X	Y
24	-0.1	Late	31.324	-0.856	34.917	1152	64.661	314000	335000
20	-0.407	Late	56.426	-0.131	13.889	7	665.964	573000	144000
24	-0.1	Early	0.766	-1.404	-68.43	2743	18.087	460000	303000
32	0.426	Late	0.055	0	-53.996	0	48.811	519000	170000
12	-1.171	Early	6.128	-0.196	49.969	8395	141.795	349000	106000
58	1.692	Late	1.666	0	-28.997	2889	48.863	485000	146000
11	-1.288	Late	21.875	1.322	35.3	5247	352.439	371000	254000
9	-1.545	Early	14.255	0.178	41.81	11458	332.158	384000	212000
12	-1.171	Early	78.387	4.822	-0.8	11769	782.873	462000	439000
10	-1.412	Early	27.744	12.224	-27.738	8214	350.238	591000	163000
37	0.711	Early	0.453	0	-53.587	3909	31.32	489000	159000
51	1.396	Late	4.894	-0.327	32.318	3656	94.428	470000	184000
33	0.485	Late	24.068	1.147	-36.895	884	281.906	323000	126000
22	-0.249	Early	1.532	0	51.816	72	113.142	250000	77000
33	0.485	Early	3.121	0	22.356	9507	137.724	286000	661000
4	-2.421	Early	86.675	4.796	2.781	13610	486.281	615000	286000
18	-0.576	Early	59.742	6.12	17.027	10535	768.199	442000	177000
19	-0.49	Early	5.169	0	10.681	881	25.786	265000	315000
6	-2.014	Early	50.781	1.304	10.445	2828	597.201	495000	196000
26	0.041	Late	1.683	0	-35.472	0	40.121	416000	93000
21	-0.327	Late	64.512	-2.254	8.638	56	822.051	512000	250000
69	2.114	Late	0.045	0	-39.751	2758	51.628	542000	174000
28	0.175	Early	5.807	0.354	2.908	1122	135.24	501000	167000
32	0.426	Late	8.92	-0.3	30.109	251	216.021	368000	227000
10	-1.412	Early	79.282	10.501	7.521	11537	632.449	584000	254000
20	-0.407	Early	15.004	-0.386	37.668	869	455.15	215000	558000
21	-0.327	Late	9.805	-0.188	19.037	0	178.342	338000	161000
25	-0.028	Late	0	-3.39	-68.626	36	45.707	521000	181000
29	0.24	Late	3.553	0	32.72	294	135.083	304000	251000
39	0.818	Late	8.823	-3.616	-38.067	1279	297.096	384000	254000
7	-1.843	Early	76.823	5.588	2.814	12424	582.163	595000	266000
6	-2.014	Early	71.247	13.629	10.669	13855	468.291	627000	255000
27	0.109	Late	46.539	5.521	26.589	3891	540	386000	186000
15	-0.854	Early	15.339	2.51	8.054	4492	317.149	475000	182000
34	0.543	Early	4.234	-3.608	29.018	2821	70.171	541000	134000
23	-0.173	Late	0.055	0	-63.517	0	39.643	520000	169000
13	-1.06	Early	62.382	5.432	4.382	12418	540.276	594000	266000
13	-1.06	Late	10.801	0.208	13.751	738	233.736	532000	139000

27	0.109	Late	8.091	-9.232	-56.865	6519	39.47	546000	261000
35	0.6	Late	1.164	0	-48.817	199	67.016	526000	162000
13	-1.06	Early	29.573	3.706	32.769	9130	431.662	373000	247000
20	-0.407	Late	53.982	0.747	9.706	145	439.044	587000	213000
8	-1.688	Late	35.883	-4.575	6.052	0	465.25	387000	259000
10	-1.412	Early	46.892	-0.246	-19.112	10087	492.363	510000	320000
10	-1.412	Late	75.948	-10.656	3.596	7158	745.792	505000	349000
14	-0.954	Late	8.516	-4.126	-17.89	1424	156.572	539000	139000
30	0.303	Late	0	0	14.209	73	16.597	496000	499000
17	-0.665	Late	23.162	5.448	4.696	10602	264.088	491000	149000
10	-1.412	Early	20.316	3.812	44.813	6555	528.368	329000	250000
12	-1.171	Late	27.244	3.461	30.849	11950	397.514	594000	140000
12	-1.171	Early	27.269	-1.637	23.637	11829	338.473	607000	155000
17	-0.665	Late	71.491	-34.481	6.483	17	1095.166	319000	732000
26	0.041	Early	35.979	-26.559	13.854	4728	527.934	375000	821000
11	-1.288	Late	76.768	1.629	9.085	369	563.875	572000	243000
10	-1.412	Early	73.392	1.607	2.823	12467	596.413	557000	235000
34	0.543	Late	18.529	1.185	3.005	287	262.254	446000	165000
17	-0.665	Early	31.461	-0.847	21.538	8509	434.406	472000	180000
15	-0.854	Late	59.128	-9.26	16.743	0	851.862	339000	707000
33	0.485	Late	23.534	-3.945	-9.921	227	336.439	479000	266000
45	1.12	Late	29.437	-1.687	-8.15	724	363.524	516000	215000
73	2.256	Late	27.571	-1.235	-0.257	332	338.317	531000	213000
10	-1.412	Early	77.479	6.12	7.502	12672	675.014	475000	376000
9	-1.545	Early	59.778	2.79	4.963	12746	409.375	470000	375000
8	-1.688	Early	42.223	3.119	21.084	8012	728.015	566000	150000
12	-1.171	Early	69.104	-3.366	13.902	13369	799.488	401000	242000
24	-0.1	Early	4.188	-10.382	-36.144	247	281.864	511000	164000
10	-1.412	Early	44.392	-0.905	7.841	12643	405.41	522000	215000
25	-0.028	Late	33.234	-7.384	29.241	6015	564.455	402000	840000
15	-0.854	Early	30.062	-10.209	15.536	11212	210.203	273000	44000
59	1.733	Late	20.668	-4.047	-12.953	652	261.007	485000	187000
12	-1.171	Early	0.284	0	-29.194	8	76.461	516000	167000
11	-1.288	Early	25.179	-1.437	25.912	12098	481.03	521000	148000
45	1.12	Late	2.924	-0.829	18.333	5151	150.941	501000	144000
11	-1.288	Early	74.371	8.172	9.225	12361	607.326	637000	272000
8	-1.688	Early	83.787	-5.312	-0.473	13190	533.807	596000	275000
7	-1.843	Early	49.903	1.44	4.957	12885	556.657	501000	207000
14	-0.954	Early	4.81	0.42	-47.674	8483	111.457	613000	167000
39	0.818	Late	71.571	-13.582	0.186	15	746.187	451000	224000
10	-1.412	Early	59.819	0.361	13.259	4760	742.432	404000	249000
34	0.543	Late	35.623	-8.858	10.55	2074	255.8	286000	63000
40	0.871	Early	58.483	-2.055	-14.765	2012	472.916	506000	345000
31	0.365	Early	7.506	-7.191	-53.023	155	88.864	290000	65000
24	-0.1	Early	0	0	-59.908	305	31.131	413000	280000
9	-1.545	Late	1.661	-0.128	33.215	13001	164.706	415000	115000

17	-0.665	Late	32.69	1.612	27.386	1004	298.051	499000	126000
17	-0.665	Early	0.583	-3.992	-51.32	4708	67.581	483000	238000
21	-0.327	Early	2.846	0	-44.396	72	31.403	459000	100000
10	-1.412	Early	7.71	1.19	-52.787	5254	74.978	615000	246000
26	0.041	Late	0	0	-60.689	0	15.39	539000	188000
33	0.485	Late	39.744	2.137	4.333	4602	390.368	619000	239000
30	0.303	Late	2.626	-3.324	21.144	428	114.893	369000	610000
10	-1.412	Early	26.726	0.565	28.995	10109	466.396	330000	281000
9	-1.545	Early	54.397	4.075	6.287	12539	512.177	518000	211000
49	1.306	Late	11.42	1.311	-31.571	488	176.147	468000	106000
53	1.483	Late	14.022	3.95	-8.029	154	206.507	544000	195000
23	-0.173	Early	21.34	-4.281	5.959	6453	239.417	397000	228000
9	-1.545	Early	2.024	-0.682	9.865	9398	161.801	516000	150000
35	0.6	Late	41.814	-4.9	25.285	3712	405.287	602000	237000
23	-0.173	Early	35.082	-4.026	23.154	7512	375.563	583000	161000
28	0.175	Late	4.745	0.046	-43.535	41	85.287	411000	278000
6	-2.014	Early	40.587	3.704	29.462	12536	578.543	364000	233000
14	-0.954	Early	37.192	2.663	-30.369	10363	337.677	582000	215000
8	-1.688	Early	20.67	-1.944	-39.041	2455	267.983	547000	211000
5	-2.204	Early	10.341	-1.799	40.285	13185	480.525	374000	229000
35	0.6	Late	0.597	4.72	-50.137	3304	38.218	333000	386000
7	-1.843	Early	37.866	2.732	26.703	12596	418.236	332000	125000
9	-1.545	Early	63.058	10.906	10.051	12667	1017.772	361000	227000
11	-1.288	Early	40.251	-1.355	28.031	13034	314.063	297000	103000
12	-1.171	Late	0.403	0	23.482	0	64.575	276000	894000
12	-1.171	Early	12.154	6.258	29.401	11133	362.661	371000	222000
11	-1.288	Early	59.916	-3.279	6.333	12852	539.464	443000	438000
7	-1.843	Early	27.685	4.197	30.253	13606	306.159	316000	234000
23	-0.173	Late	4.947	0	3.839	231	103.567	294000	181000
27	0.109	Late	32.788	0.606	13.093	369	391.669	336000	124000
13	-1.06	Late	14.774	3.688	26.798	0	282.336	502000	131000
8	-1.688	Early	54.625	4.714	-11.446	13392	424.089	498000	364000
14	-0.954	Early	16.792	-0.52	15.548	1285	251.341	209000	65000
21	-0.327	Early	11.05	-4.703	-33.501	476	93.806	620000	310000
28	0.175	Early	2.777	-5.028	-41.188	4203	53.948	453000	108000
12	-1.171	Early	30.672	4.593	34.513	11202	476.422	398000	255000
8	-1.688	Early	50.127	-4.961	24.888	12746	396.876	413000	226000
10	-1.412	Early	21.738	4.515	-17.288	9881	398.329	436000	210000
16	-0.757	Early	2.199	-2.213	-44.646	7799	64.693	455000	206000
25	-0.028	Late	0.045	0	-6.327	0	35.693	410000	418000
31	0.365	Late	2.532	1.269	31.52	720	102.025	416000	501000
29	0.24	Late	15.018	-4.682	1.029	1583	195.302	418000	498000
21	-0.327	Early	41.651	3.236	28.503	8818	662.531	376000	222000
43	1.022	Late	2.236	-0.041	31.777	1907	132.953	347000	153000
15	-0.854	Early	24.523	-17.481	-35.528	9539	363.482	458000	107000
23	-0.173	Late	15.786	-6.878	-45.801	0	274.79	517000	207000

28	0.175	Early	2.413	-4.645	-48.326	454	62.91	509000	175000
15	-0.854	Early	26.983	2.237	-19.289	4858	283.032	524000	221000
61	1.812	Late	57.1	-4.686	8.643	4578	736.486	502000	215000
36	0.656	Late	0.045	-1.411	-64.763	0	20.755	523000	186000
11	-1.288	Early	58.532	9.735	15.525	13035	793.768	423000	187000
13	-1.06	Early	44.809	-15.411	4.74	12244	361.649	568000	162000
11	-1.288	Early	8.495	1.83	45	7053	291.582	393000	334000
20	-0.407	Late	8.615	-0.096	34.827	3031	284.114	538000	128000
15	-0.854	Late	0.533	-1.628	4.289	0	64.146	402000	393000
9	-1.545	Early	45.194	2.435	7.056	11808	484.924	527000	217000
19	-0.49	Early	0.622	-0.616	48.39	4604	108.183	295000	139000
18	-0.576	Early	27.16	4.049	-22.58	8490	182.636	439000	560000
11	-1.288	Early	21.437	-4.307	27.247	12476	379.211	371000	224000
12	-1.171	Early	36.516	9.182	-1.389	12396	492.252	328000	138000
14	-0.954	Early	0	0	8.678	10925	24.463	257000	354000
29	0.24	Early	0	-3.343	-40.292	184	82.556	512000	175000
10	-1.412	Early	23.924	-3.631	16.248	12183	281.514	501000	198000
21	-0.327	Late	33.002	9.471	4.796	7566	311.793	524000	205000
10	-1.412	Early	12.744	-11.992	-4.529	12387	269.373	529000	153000
11	-1.288	Early	49.038	4.376	-13.928	12889	586.455	451000	392000
42	0.973	Late	15.778	5.787	-48.511	215	188.85	517000	208000
8	-1.688	Early	62.923	-2.218	4.538	10553	789.849	393000	246000
41	0.922	Late	53.805	-2.355	5.948	2572	809.517	395000	244000
10	-1.412	Early	43.899	1.787	17.818	11901	641.775	346000	242000
18	-0.576	Early	9.2	-2.233	-20.754	9017	217.485	367000	177000
15	-0.854	Early	0.473	-11.51	-60.328	0	27.09	529000	180000
8	-1.688	Early	34.506	3.375	-29.413	9881	461.96	461000	153000
9	-1.545	Early	4.599	0.263	21.219	6286	105.013	398000	227000
40	0.871	Late	14.765	-2.101	26.554	16	317.666	506000	158000
36	0.656	Late	0.389	-3.999	19.737	1011	98.648	461000	85000
43	1.022	Late	0.249	0	-12.785	1917	88.41	516000	168000
44	1.072	Late	0.383	-0.169	-20.569	16	64.78	521000	160000
24	-0.1	Early	9.5	-3.121	-11.48	6038	176.44	552000	154000
12	-1.171	Early	67.692	8.934	8.546	11549	525.281	303000	141000
37	0.711	Late	78.861	-3.756	6.553	5559	630.536	495000	353000
22	-0.249	Early	0	-0.65	-21.329	1644	63.379	443000	104000
26	0.041	Early	0.594	1.513	-10.928	259	65.707	491000	169000
30	0.303	Late	54.237	8.702	20.058	29	613.704	465000	130000
8	-1.688	Late	7.003	-0.771	7.312	0	284.382	227000	643000
7	-1.843	Early	28.338	-6.28	33.433	13522	406.767	416000	126000
35	0.6	Late	69.104	-3.366	13.902	2847	799.488	401000	242000
25	-0.028	Late	13.334	-9.342	-36.933	0	218.073	467000	174000
10	-1.412	Late	58.58	-2.016	-14.701	13096	472.916	506000	345000
6	-2.014	Early	62.009	-4.857	11.359	4158	914.928	520000	225000
20	-0.407	Late	25.167	-1.425	25.861	540	481.03	521000	148000
17	-0.665	Late	52.823	-9.899	7.639	1302	161.207	159000	28000

14	-0.954	Early	4.924	-1.021	38.064	425	147.595	354000	173000
15	-0.854	Late	73.428	1.73	2.802	2425	596.413	557000	235000
7	-1.843	Early	3.441	1.201	48.844	12191	196.204	334000	221000
23	-0.173	Late	28.512	-6.762	33.648	1841	406.767	416000	126000
17	-0.665	Late	45.194	2.435	7.056	0	484.924	527000	217000
34	0.543	Late	26.074	0.11	32.894	649	445.707	526000	146000
11	-1.288	Early	19.168	-0.859	35.038	11934	234.796	334000	127000
20	-0.407	Late	1.4	0	-32.815	15	28.725	489000	158000
29	0.24	Late	59.655	-9.491	17.079	329	768.199	442000	177000
22	-0.249	Early	6.029	0	19.05	355	82.114	257000	561000
10	-1.412	Early	73.147	-11.325	13.639	12747	1027.99	375000	774000
17	-0.665	Late	86.979	0.379	0.802	10821	875.764	471000	432000
25	-0.028	Late	29.425	3.653	32.81	388	431.662	373000	247000
11	-1.288	Early	56.569	7.101	12.431	10202	509.711	589000	227000
21	-0.327	Late	0.254	0	-54.596	13	42.563	542000	178000
32	0.426	Late	65.306	3.073	2.629	3335	471.595	470000	343000
11	-1.288	Early	14.743	-0.07	-44.834	13029	123.463	504000	429000
13	-1.06	Late	63.451	2.916	2.944	9	617.932	593000	223000
23	-0.173	Early	10.978	6.831	-26.05	9868	132.018	325000	390000
26	0.041	Late	50.01	1.385	4.834	675	556.657	501000	207000
29	0.24	Late	46.888	2.744	20.008	342	519.834	285000	180000
9	-1.545	Early	55.68	0.699	-0.505	12227	517.252	428000	209000
21	-0.327	Late	59.778	2.79	4.963	20	409.375	470000	375000
36	0.656	Late	42.532	2.518	21.122	124	705.045	566000	149000
14	-0.954	Early	69.934	-30.682	15.029	11030	1107.356	377000	829000
18	-0.576	Early	8.71	-0.187	34.787	10888	278.007	538000	128000
20	-0.407	Late	15.547	-4.415	18.981	0	111.016	170000	45000
11	-1.288	Early	0	0	16.921	12154	84.326	490000	143000
39	0.818	Late	0.139	0	-69.579	0	17.489	535000	176000
16	-0.757	Late	32.801	-5.739	32.104	9430	287.196	234000	64000
46	1.167	Late	0	0	50.543	4	115.02	382000	556000
24	-0.1	Late	0	0	19.047	0	15.165	400000	429000
12	-1.171	Early	0.199	0	-51.946	0	46.042	524000	169000
18	-0.576	Late	32.697	-12.61	6.551	12	326.735	576000	289000
33	0.485	Late	47.572	-29.294	21.897	3416	695.34	372000	825000
6	-2.014	Early	69.294	-1.148	17.042	12249	662.548	469000	229000
30	0.303	Late	0.607	0	31.662	2793	83.212	401000	446000
15	-0.854	Early	26.216	-9.212	9.351	1096	93.444	212000	51000
13	-1.06	Late	2.338	0	12.204	0	68.395	313000	188000
10	-1.412	Late	27.033	-1.724	41.2	9421	311.974	369000	112000
22	-0.249	Early	0	0	21.894	55	18.012	400000	427000
44	1.072	Late	79.818	6.548	6.347	180	1070.149	456000	187000
15	-0.854	Late	67.454	-23.825	5.814	12422	540.166	430000	144000
6	-2.014	Early	62.343	-8.512	14.788	10633	603.658	453000	221000
34	0.543	Late	6.029	0	19.05	185	82.114	257000	561000
13	-1.06	Late	30.997	-1.225	20.175	85	285.179	337000	447000

33	0.485	Late	59.148	1.784	8.487	5422	578.249	435000	295000
7	-1.843	Late	6.371	-2.089	18.059	0	155.184	320000	474000
23	-0.173	Late	46.926	-12.205	23.254	3314	486.618	387000	115000
38	0.765	Late	37.376	-0.509	-7.922	0	402.043	524000	219000
34	0.543	Late	20.85	-3.436	5.064	775	239.417	397000	228000
23	-0.173	Late	26.726	0.565	28.995	0	466.396	330000	281000
33	0.485	Late	16.517	-1.564	-37.065	0	185.702	418000	188000
22	-0.249	Late	17.722	5.626	-33.325	9116	197.298	544000	259000
18	-0.576	Late	14.178	-0.638	48.111	11284	197.481	341000	107000
34	0.543	Late	6.117	0.69	49.868	33	141.795	349000	106000
13	-1.06	Late	27.269	-1.637	23.637	26	338.473	607000	155000
63	1.89	Late	2.323	2.034	-38.872	2285	109.477	528000	196000
10	-1.412	Early	22.955	4.969	13.998	12738	269.056	315000	142000
31	0.365	Late	0	0	48.653	7	105.852	402000	350000
18	-0.576	Late	8.822	-4.737	21.887	30	137.625	608000	160000
22	-0.249	Late	8.507	3.682	-26.468	0	255.804	479000	169000
24	-0.1	Late	79.105	-17.133	9.519	111	731.517	405000	220000
21	-0.327	Late	27.695	-8.378	24.881	2625	556.188	476000	151000
27	0.109	Late	13.43	6.373	-29.094	986	222.863	374000	163000
30	0.303	Late	1.403	0	-13.373	152	94.329	504000	163000
25	-0.028	Late	24.711	0.155	25.55	1648	409.501	394000	173000
23	-0.173	Late	75.199	6.809	5.67	2170	730.207	535000	231000
30	0.303	Late	15.339	2.51	8.054	1213	317.149	475000	182000
25	-0.028	Late	69.969	-30.486	15.059	2694	1107.356	377000	829000
29	0.24	Late	0	-2.605	-42.969	0	47.952	542000	174000
38	0.765	Late	2.233	0	17.423	283	73.667	294000	566000
38	0.765	Late	3.14	-0.667	23.262	144	66.04	352000	206000
15	-0.854	Late	2.407	-4.6	-48.358	0	61.09	509000	175000
43	1.022	Late	65.222	-18.106	16.447	130	669.209	395000	193000
32	0.426	Late	25.285	6.608	-10.212	0	411.768	401000	162000
37	0.711	Late	8.451	-29.517	-8.532	35	326.382	266000	843000
22	-0.249	Late	23.121	1.115	-40.891	3377	208.371	458000	379000
32	0.426	Late	1.767	-2.298	-6.765	906	78.845	352000	492000
52	1.44	Late	14.193	10.998	-51.178	647	431.268	457000	106000
19	-0.49	Late	0.144	0	-51.774	14	34.488	526000	168000
22	-0.249	Late	0.409	0	-53.676	0	31.32	489000	159000
43	1.022	Late	67.279	-20.929	6.958	625	975.323	347000	735000
41	0.922	Late	0.982	0	47.916	109	125.686	378000	444000
48	1.261	Late	0.781	-3.157	-28.273	7	97.2	550000	173000
9	-1.545	Late	0	0	6.912	0	85.821	185000	731000
37	0.711	Late	0.159	0	-54.407	3569	18.555	541000	180000
19	-0.49	Late	34.413	3.762	24.407	11240	411.912	487000	117000
58	1.692	Late	0.736	-2.761	19.637	4119	166.832	260000	873000
20	-0.407	Late	85.079	2.176	5.946	7333	692.92	545000	234000
21	-0.327	Late	9.2	-2.233	-20.754	363	217.485	367000	177000
24	-0.1	Late	14.305	2.126	6.23	317	230.444	355000	180000

27	0.109	Late	0.289	0	-53.734	257	37.392	534000	173000
17	-0.665	Late	10.159	-0.995	44.505	0	172.527	251000	263000
29	0.24	Late	89.689	4.792	1.406	2470	614.08	601000	267000
26	0.041	Late	0.1	0	-65.638	23	17.185	525000	170000
11	-1.288	Late	5.377	-0.019	34.729	21	170.941	348000	457000
33	0.485	Late	4.513	-9.262	-19.096	0	289.472	512000	164000
38	0.765	Late	66.672	9.672	1.782	2009	608.892	471000	449000
33	0.485	Late	6.182	0.774	-4.732	15	262.947	500000	191000
39	0.818	Late	3.863	-2.571	46.192	31	177.059	348000	528000
14	-0.954	Late	12.918	-9.051	-46.193	4135	129.406	454000	319000
21	-0.327	Late	5.004	0	4.005	0	63.931	496000	165000
23	-0.173	Late	44.952	3.311	1.926	200	395.415	462000	337000
8	-1.688	Late	60.015	-6.926	4.69	11251	499.525	474000	405000
23	-0.173	Late	77.447	6.127	7.496	175	675.014	475000	376000
34	0.543	Late	44.525	10.412	11.208	786	584.562	499000	106000
38	0.765	Late	3.854	0.114	50.58	4639	135.117	268000	258000
28	0.175	Late	8.635	-0.128	34.789	33	284.114	538000	128000
33	0.485	Late	1.835	-8.664	-48.958	647	45.914	544000	181000
12	-1.171	Late	54.811	-1.431	18.23	415	813.148	476000	217000
23	-0.173	Late	62.392	-8.262	14.761	8	603.658	453000	221000
16	-0.757	Late	69.472	-1.242	17.074	111	662.548	469000	229000
19	-0.49	Late	0.055	0	-54.72	33	59.728	516000	174000
20	-0.407	Late	65.861	-54.68	0.212	0	1383.854	302000	860000
19	-0.49	Early	21.213	2.038	-6.19	34	316.15	508000	204000
24	-0.1	Late	30.559	3.266	36.029	912	244.964	401000	571000
54	1.526	Late	12.967	-1.829	26.786	860	264.4	504000	157000
45	1.12	Late	25.706	3.413	-41.477	273	316.689	493000	100000
26	0.041	Late	20.271	3.798	44.85	21	528.368	329000	250000
87	2.718	Late	18.107	-29.912	-29.006	4238	219.497	621000	245000
31	0.365	Late	14.548	-1.465	-23.643	3170	295.442	451000	106000
35	0.6	Late	6.69	-20.137	27.405	181	259.92	349000	844000
18	-0.576	Late	76.094	2.375	7.441	319	660.374	536000	360000
15	-0.854	Late	30.062	-10.209	15.536	0	210.203	273000	44000
70	2.15	Late	50.303	0.185	18.418	487	667.68	418000	236000
36	0.656	Late	14.743	-0.07	-44.834	2392	123.463	504000	429000
13	-1.06	Late	82.11	10.473	4.767	12086	534.394	597000	260000
18	-0.576	Late	74.03	-20.662	9.013	13	972.993	343000	738000
20	-0.407	Late	28.041	-4.398	28.542	22	556.365	289000	723000
28	0.175	Late	5.378	-5.774	-29.953	0	110.147	571000	158000
49	1.306	Late	0.015	0	-52.821	11	58.187	516000	173000
18	-0.576	Late	61.323	4.141	14.951	459	449.209	462000	482000
41	0.922	Late	3.856	0.089	-42.315	35	52.748	392000	389000
33	0.485	Late	0.483	-0.609	38.065	61	140.871	322000	359000
19	-0.49	Late	2.194	-0.035	33.861	4501	157.347	232000	551000
38	0.765	Late	0.209	0.6	-45.88	1020	32.459	439000	385000
25	-0.028	Late	0	-3.339	-40.339	0	82.556	512000	175000

37	0.711	Late	48.815	-3.469	-16.997	399	544.896	524000	234000
24	-0.1	Late	71.289	5.596	5.719	282	533.608	598000	268000
32	0.426	Late	62.923	-2.218	4.538	4450	789.849	393000	246000
11	-1.288	Late	49.09	4.427	-13.837	7484	586.455	451000	392000
24	-0.1	Late	0	0	0.729	0	18.371	290000	193000
46	1.167	Late	0.07	0	-36.855	0	36.544	527000	188000
28	0.175	Late	0.089	-1.111	-54.76	29	23.52	518000	185000
35	0.6	Late	1.403	0	-13.414	115	94.329	504000	163000
33	0.485	Late	37.108	-3.626	18.104	156	511.916	401000	195000
30	0.303	Late	32.962	1.548	27.296	3224	298.051	499000	126000
27	0.109	Late	13.776	6.159	22.13	46	321.287	550000	163000
31	0.365	Late	59.686	1.982	11.107	1667	400.122	618000	278000
37	0.711	Late	0.03	0	-40.555	0	14.772	432000	386000
13	-1.06	Late	3.524	-1.55	42.442	0	152.44	198000	214000
14	-0.954	Late	0	-0.777	-42.15	328	51.842	514000	171000
28	0.175	Late	1.055	-0.288	33.659	7197	147.098	415000	115000
33	0.485	Late	4.768	0.459	-48.062	3274	111.457	613000	167000
62	1.851	Late	78.412	-0.83	-0.724	1482	782.873	462000	439000
29	0.24	Late	8.007	2.172	-53.623	522	50.615	426000	568000
35	0.6	Late	41.372	3.992	29.608	64	566.868	364000	234000
33	0.485	Late	6.361	-3.633	54.778	439	210.642	227000	332000
29	0.24	Late	59.821	4.951	11.867	102	390.102	587000	327000
23	-0.173	Late	24.918	5.361	29.82	702	243.412	451000	480000
13	-1.06	Late	62.627	-21.423	7.112	5786	561.635	408000	145000
21	-0.327	Late	84.792	4.053	1.185	689	862.54	458000	428000
25	-0.028	Late	29.366	4.247	35.158	46	502.944	400000	158000
28	0.175	Late	39.212	-17.336	1.833	7187	743.852	303000	686000
13	-1.06	Late	41.651	3.236	28.503	0	662.531	376000	222000
33	0.485	Late	18.129	5.625	-33.003	335	197.298	544000	259000
29	0.24	Early	5.94	-0.479	39.285	0	113.538	211000	208000
47	1.214	Late	5.085	-2.116	-11.455	179	162.349	534000	125000
29	0.24	Late	5.673	-4.963	-14.273	0	122.762	356000	175000
29	0.24	Late	0.055	0	-53.884	0	48.811	519000	170000
40	0.871	Late	45.212	11.133	-11.943	2915	548.225	462000	191000
11	-1.288	Late	3.24	-8.833	-55.889	0	85.673	378000	395000
38	0.765	Late	1.208	-0.04	33.949	333	114.844	557000	119000
60	1.773	Late	28.594	-4.468	2.49	32	259.791	614000	133000
26	0.041	Late	12.132	-1.607	26.487	10464	277.751	466000	244000
30	0.303	Late	7.465	-8.776	39.544	0	208.97	372000	168000
45	1.12	Late	39.685	3.164	11.441	33	491.603	530000	213000
25	-0.028	Late	7.741	-0.274	51.64	4491	260.426	400000	186000
29	0.24	Late	23.121	1.115	-40.891	332	208.371	458000	379000
20	-0.407	Late	12.154	6.258	29.401	55	362.661	371000	222000
35	0.6	Late	0.169	-3.974	-55.939	656	30.16	535000	191000
12	-1.171	Late	6.846	-2.457	45.782	2257	169.62	196000	224000
43	1.022	Late	66.58	-2.083	16.457	232	858.016	410000	157000

49	1.306	Late	42.164	-2.365	5.521	6137	621.09	473000	151000
17	-0.665	Late	36.703	-2.747	28.044	58	572.622	393000	176000
18	-0.576	Late	65.455	1.009	0.806	88	367.63	616000	303000
25	-0.028	Late	0	0	25.818	120	75.289	395000	549000
38	0.765	Late	0	0	32.87	7	68.276	391000	585000
24	-0.1	Late	0.07	0	-21.127	4036	77.984	534000	158000
24	-0.1	Late	3.382	0.162	6.151	306	94.736	411000	553000
26	0.041	Late	26.492	3.6	37.613	808	471.584	399000	158000
23	-0.173	Late	4.149	-0.002	49.634	173	172.746	256000	257000
50	1.351	Late	15.117	-6.075	-3.112	1154	295.612	329000	378000
18	-0.576	Late	85.455	2.785	-1.151	0	1055.564	528000	322000
21	-0.327	Late	15.717	-0.777	44.882	2002	239.738	363000	110000
43	1.022	Late	71.046	7.719	5.101	162	579.826	478000	379000
18	-0.576	Late	34.761	1.987	24.432	9202	426.903	382000	132000
27	0.109	Late	11.966	0	-30.444	100	70.244	292000	181000
29	0.24	Late	63.016	10.915	9.954	0	1017.772	361000	227000
24	-0.1	Late	1.602	-0.73	31.556	0	65.959	252000	70000
15	-0.854	Late	0.255	0	32.493	0	52.61	409000	436000
36	0.656	Late	39.046	-7.421	25.493	268	254.676	251000	137000
4	-2.421	Late	60.632	10.015	-7.844	17	423.296	529000	403000
27	0.109	Late	0.533	0	16.703	0	44.233	187000	889000
6	-2.014	Late	2.014	0	-4.993	0	29.471	309000	196000
36	0.656	Late	73.141	-14.232	-2.946	401	1034.031	517000	241000
20	-0.407	Late	0	0	11.153	44	20.805	395000	430000
43	1.022	Late	62.201	-5.235	11.485	232	914.928	520000	225000
27	0.109	Late	85.194	12.442	-0.015	3983	649.317	554000	318000
26	0.041	Late	21.844	-17.58	-20.531	2131	282.714	475000	257000
47	1.214	Late	0.622	-0.615	48.399	561	108.183	295000	139000
53	1.483	Late	0	0	-66.619	0	11.454	527000	172000
34	0.543	Late	4.383	-0.903	-0.786	77	155.133	195000	215000
16	-0.757	Late	11.81	2.262	19.857	0	253.446	433000	316000
4	-2.421	Late	16.359	13.498	-46.789	2	247.052	436000	290000
20	-0.407	Late	68.688	-3.472	14.996	2754	459.636	422000	615000
32	0.426	Late	29.375	-9.214	15.431	153	163.736	200000	51000
57	1.652	Late	24.1	-1.29	-36.844	1350	281.906	323000	126000
28	0.175	Late	23.784	3.648	25.208	263	460.309	513000	131000
63	1.89	Late	50.627	-9.789	13.497	599	623.014	417000	160000
51	1.396	Late	0	0	-2.533	25	39.579	387000	422000
38	0.765	Late	89.724	4.759	1.404	8359	614.08	601000	267000
36	0.656	Late	56.294	-5.603	18.207	563	588.85	447000	226000
30	0.303	Late	46.541	-25.917	14.948	0	605.213	328000	863000
18	-0.576	Late	18.97	-0.909	35.025	0	249.635	334000	127000
39	0.818	Late	0.149	-2.212	-59.828	554	32.338	618000	243000
17	-0.665	Late	5.272	-0.245	12.729	250	130.56	360000	206000
45	1.12	Late	53.609	2.654	4.362	2740	402.221	433000	543000
44	1.072	Late	35.986	-4.789	1.268	93	465.25	387000	259000

54	1.526	Late	14.548	-1.465	-23.643	6007	295.442	451000	106000
37	0.711	Late	10.644	-1.277	40.463	51	219.561	387000	375000
18	-0.576	Late	28.989	6.272	21.363	0	465.822	547000	155000
27	0.109	Late	0	0	-51.074	92	6.726	266000	193000
47	1.214	Late	0.662	0	52.336	61	187.897	243000	374000
31	0.365	Late	1.353	0	-5.593	24	60.551	495000	157000
40	0.871	Late	2.637	0.131	-26.826	59	135.288	522000	105000
38	0.765	Late	0.597	4.819	-50.237	189	38.218	333000	386000
34	0.543	Late	0.706	-0.858	17.952	984	78.007	278000	376000
51	1.396	Late	41.964	-1.629	0.103	773	574.863	449000	192000
46	1.167	Late	4.049	8.194	-54.959	31	101.898	547000	167000
38	0.765	Late	0.681	-0.437	41.584	42	127.774	401000	347000
58	1.692	Late	74.792	-14.334	13.115	558	476.067	394000	111000
34	0.543	Late	35.996	2.089	33.787	140	612.528	344000	324000
27	0.109	Late	51.61	-9.305	18.275	0	406.891	421000	221000
41	0.922	Late	12.428	-15.002	16.581	2674	274.465	355000	634000
31	0.365	Late	23.994	3.481	23.469	348	330.001	405000	262000
31	0.365	Late	25.997	-3.594	-0.815	0	560.74	489000	148000
31	0.365	Late	38.393	-8.998	12.689	2982	139.933	211000	52000
78	2.427	Late	0	-17.996	-74.934	446	4.333	534000	182000
21	-0.327	Late	0	0	16.792	2	81.213	490000	143000
19	-0.49	Late	2.567	-0.041	-8.711	0	190.788	507000	167000
23	-0.173	Late	3.113	-0.692	20.033	1889	100.122	247000	74000
38	0.765	Late	12.792	-1.146	-30.314	1878	140.23	481000	152000
32	0.426	Late	7.543	-2.863	5.837	39	263.331	483000	167000
39	0.818	Late	44.773	8.488	-10.985	165	390.738	432000	411000
18	-0.576	Late	75.109	8.003	8.593	1184	436.417	622000	291000
37	0.711	Late	0.433	0	33.173	4	85.873	379000	586000
37	0.711	Late	1.105	2.212	-41.8	87	57.817	521000	163000
32	0.426	Late	75.782	14.853	5.745	9	685.521	602000	193000
66	2.004	Late	0	-1.122	-67.894	869	19.269	509000	432000
52	1.44	Late	18.68	-10.27	17.316	3451	184.47	492000	188000
18	-0.576	Late	9.256	5.805	-38.466	0	361.753	458000	105000
12	-1.171	Late	1.834	0	48.725	63	135.445	274000	233000
34	0.543	Late	0.294	0	21.258	174	64.958	276000	895000
37	0.711	Late	14.78	0.111	40.238	5341	413.26	520000	140000
55	1.568	Late	50.041	-4.935	24.938	30	396.876	413000	226000
34	0.543	Late	34.631	12.259	-22.05	19	497.037	523000	223000
18	-0.576	Late	35.782	-26.435	14.145	5841	527.934	375000	821000
48	1.261	Late	2.904	-0.375	8.662	786	89.385	327000	193000
45	1.12	Late	7.306	-0.81	-38.346	69	133.344	333000	160000
35	0.6	Late	0	0	21.771	57	18.012	400000	427000
30	0.303	Late	53.198	-10.116	7.148	3592	161.207	159000	28000
54	1.526	Late	4.73	-1.547	0.666	324	160.416	522000	196000
53	1.483	Late	35.981	2.369	22.291	1270	326.509	456000	319000
16	-0.757	Late	10.87	0	26.278	75	183.289	338000	161000

43	1.022	Late	22.368	0.77	37.105	2609	239.948	345000	550000
30	0.303	Late	7.846	-4.38	-32.555	0	159.557	469000	111000
43	1.022	Late	14.466	0.386	23.888	696	115.21	245000	71000
50	1.351	Late	51.731	-16.432	-19.434	412	658.237	521000	210000
54	1.526	Late	40.57	0.392	15.84	2571	657.312	550000	165000
23	-0.173	Late	4.452	-0.736	21.136	68	91.497	367000	424000
52	1.44	Late	73.892	5.692	-4.873	381	641.826	474000	371000
46	1.167	Late	1.937	-2.331	-9.509	1289	70.452	454000	108000
31	0.365	Late	42.28	-1.941	10.765	0	99.586	171000	15000
39	0.818	Late	21.14	3.88	-2.6	177	320.365	392000	163000
34	0.543	Late	35.294	2.187	24.799	1112	618.771	370000	240000
30	0.303	Late	33.726	1.965	33.969	677	611.474	379000	223000
45	1.12	Late	29.225	-0.734	16.971	26	343.035	464000	175000
84	2.623	Late	28.998	-2.396	-11.911	1238	563.599	489000	148000
33	0.485	Late	0.532	-0.148	40.355	5806	164.648	555000	129000
48	1.261	Late	7.602	-1.869	-29.227	217	149.853	552000	156000
23	-0.173	Late	0	0	13.55	21	39.051	393000	425000
44	1.072	Late	11.427	9.018	-5.984	699	220.674	332000	158000
51	1.396	Late	24.523	-17.481	-35.528	4465	363.482	458000	107000
35	0.6	Late	40.097	-7.287	2.958	2451	504.562	469000	383000
34	0.543	Late	25.402	0.271	23.403	1475	345.352	394000	188000
13	-1.06	Late	2.338	0	12.204	0	68.395	313000	188000
26	0.041	Late	3.923	-10.1	27.566	0	235.825	352000	809000
45	1.12	Late	66.638	-7.034	6.568	2173	452.099	514000	420000
32	0.426	Late	22.837	-10.233	-21.826	93	110.97	292000	55000
34	0.543	Late	57.357	1.135	1.84	353	737.335	403000	163000
40	0.871	Late	47.16	-2.61	3.214	1187	493.805	458000	225000
25	-0.028	Late	58.586	-7.776	5.49	512	568.168	594000	199000
40	0.871	Late	0	0	12.352	110	66.266	490000	142000
61	1.812	Late	44.392	9.115	4.358	8154	487.497	527000	216000
50	1.351	Late	4.23	-3.672	28.643	155	70.171	541000	134000
78	2.427	Late	0.005	0	-42.882	245	46.838	544000	171000
21	-0.327	Late	48.896	2.247	18.965	20	576.895	466000	138000
45	1.12	Late	0.259	-0.779	-64.824	0	51.696	537000	184000
59	1.733	Late	0	0	-12.835	104	36.207	278000	382000
62	1.851	Late	0.801	-0.993	-68.436	227	18.087	460000	303000
47	1.214	Late	6.97	-0.818	7.083	795	284.382	227000	643000
62	1.851	Late	7.483	-0.01	22.288	2678	226.25	335000	158000
27	0.109	Late	16.074	5.983	33.05	0	499.01	368000	207000
52	1.44	Late	0.857	0	-38.148	570	7.241	245000	54000
37	0.711	Late	0	-0.032	39.248	211	95.293	345000	523000
44	1.072	Late	55.759	0.713	-0.597	127	553.535	428000	209000
27	0.109	Late	9.029	-1.895	41.205	310	107.354	225000	67000
30	0.303	Late	21.98	-3.756	34.085	3491	228.711	237000	65000
35	0.6	Late	21.551	7.249	-14.25	1248	278.245	440000	422000
48	1.261	Late	32.156	-0.829	21.831	1390	434.406	472000	180000

34	0.543	Late	0.706	0	31.906	1336	83.212	401000	446000
36	0.656	Late	0.099	0	-56.198	7233	25.614	318000	181000
26	0.041	Late	4.202	-0.19	50.997	5	204.882	235000	377000
31	0.365	Late	29.111	-5.448	19.956	349	344.764	371000	91000
31	0.365	Late	3.497	-4.754	-49.297	1136	72.627	385000	254000
53	1.483	Late	0.055	0	-63.628	4158	39.643	520000	169000
36	0.656	Late	44.043	7.547	-18.369	709	424.62	563000	196000
18	-0.576	Late	0	0	21.082	326	33.98	395000	425000
52	1.44	Late	36.727	-3.829	21.655	3571	589.396	384000	141000
87	2.718	Late	74.468	-2.854	6.065	3445	590.609	594000	273000
46	1.167	Late	6.261	0	-28.865	202	111.42	271000	658000
56	1.61	Late	18.332	-2.519	-11.306	5074	303.946	415000	132000
54	1.526	Late	34.538	4.778	16.536	3538	446.837	499000	203000
29	0.24	Late	26.471	-4.599	34.431	1156	541.426	396000	164000
33	0.485	Late	6.615	-0.446	22.262	118	146.928	517000	153000
39	0.818	Late	4.684	-3.475	32.069	3734	107.521	470000	184000
51	1.396	Late	29.936	2.291	-30.439	1151	211.32	548000	221000
24	-0.1	Late	0	0	15.766	2049	13.318	400000	427000
36	0.656	Late	40.279	-4.581	7.6	1087	494.581	590000	292000
40	0.871	Late	8.129	0.522	-3.371	129	188.559	518000	130000
21	-0.327	Late	82.476	14.108	5.142	16	748.956	563000	209000
48	1.261	Late	0.268	0	-21.589	10	93.671	515000	167000
28	0.175	Late	67.089	-3.607	10.766	11288	512.977	510000	300000
53	1.483	Late	0.06	0	-44.341	0	29.166	520000	177000
11	-1.288	Late	2.71	0	-56.518	0	29.162	427000	435000
13	-1.06	Late	5.494	-0.253	49.556	0	165.302	355000	107000
52	1.44	Late	0.055	0	-47.298	0	71.687	516000	175000
38	0.765	Late	16.161	-4.765	-49.834	1547	396.638	502000	223000
23	-0.173	Late	11.781	0.188	16.646	0	186.191	518000	154000

Table S5. Covariates used to tests whether the occurrence of *Varroa* (binary response) or deformed wing virus (DWV – binary response) could be predicted by the species richness of foraged upon plants (SR log transformed) and the mean application rate of triazole fungicides and the herbicide glyphosate within 2 km of hives. Also included is the foliar insecticide index. Spatial locations of hives are given by X and Y coordinates (m) that have been de-resolved to the nearest 1 km to protect beekeeper anonymity.

Varroa	DWV	Season	SR_Log	Triazoles	Glyphosate	FII insecticide index	X	Y
1	0	Late	3.219	0.052	0.127	64.661	314000	335000
1	0	Late	3.497	0.13	0.428	48.811	519000	170000
1	0	Early	2.565	0.489	1.603	141.795	349000	106000
1	0	Late	4.078	0.094	0.324	48.863	485000	146000
1	0	Late	2.485	2.641	4.98	352.439	371000	254000
1	0	Early	2.565	10.755	18.521	782.873	462000	439000
0	0	Early	2.398	5.314	16.579	350.238	591000	163000
0	0	Early	3.638	0.052	0.168	31.32	489000	159000
0	0	Late	3.951	0.37	1.765	94.428	470000	184000
1	0	Early	3.135	0.661	1.289	113.142	250000	77000
1	0	Early	1.609	11.824	28.031	486.281	615000	286000
1	0	Early	2.944	7.625	30.626	768.199	442000	177000
1	0	Early	2.996	0.021	0.045	25.786	265000	315000
0	1	Early	1.946	6.732	22.063	597.201	495000	196000
0	0	Late	3.296	0.041	0.209	40.121	416000	93000
1	0	Late	3.091	10.883	35.879	822.051	512000	250000
0	0	Late	4.248	0.06	0.314	51.628	542000	174000
1	1	Late	3.497	1.329	2.965	216.021	368000	227000
0	0	Early	3.045	0.862	2.583	455.15	215000	558000
1	0	Late	3.091	0.649	1.349	178.342	338000	161000
1	0	Late	3.401	0.143	0.237	135.083	304000	251000
1	1	Late	3.689	1.362	2.378	297.096	384000	254000
1	1	Early	1.946	10.44	27.379	468.291	627000	255000
1	0	Early	2.773	1.741	8.045	317.149	475000	182000
1	0	Early	3.555	0.224	0.696	70.171	541000	134000
1	0	Late	3.178	0.13	0.404	39.643	520000	169000
0	0	Early	2.639	9.196	22.455	540.276	594000	266000
1	0	Late	2.639	1.393	4.296	233.736	532000	139000
1	0	Late	3.332	0.231	0.664	39.47	546000	261000
0	0	Late	3.584	0.163	0.494	67.016	526000	162000
0	0	Early	2.639	2.873	5.925	431.662	373000	247000
1	0	Late	3.045	7.2	25.09	439.044	587000	213000
1	1	Late	2.197	3.879	8.541	465.25	387000	259000
0	0	Late	2.398	8.756	28.669	745.792	505000	349000
0	0	Late	3.434	0.023	0.054	16.597	496000	499000
0	0	Early	2.398	3.273	5.4	528.368	329000	250000

0	0	Early	2.565	3.817	11.027	338.473	607000	155000
1	1	Early	3.296	2.644	6.399	527.934	375000	821000
0	1	Late	2.485	12.052	36.926	563.875	572000	243000
0	0	Early	2.398	11.554	37.372	596.413	557000	235000
0	0	Early	2.89	4.599	21.054	434.406	472000	180000
1	0	Late	2.773	5.825	14.944	851.862	339000	707000
1	0	Late	4.304	3.538	11.395	338.317	531000	213000
0	0	Early	2.398	12.774	26.526	675.014	475000	376000
0	0	Early	2.303	6.358	14.242	409.375	470000	375000
1	0	Early	2.197	6.79	19.921	728.015	566000	150000
1	0	Early	2.565	6.534	12.639	799.488	401000	242000
1	0	Early	3.219	1.286	3.745	281.864	511000	164000
0	0	Early	2.398	5.769	21.865	405.41	522000	215000
1	0	Late	3.258	3.246	7.149	564.455	402000	840000
1	0	Early	2.773	2.337	6.272	210.203	273000	44000
1	0	Late	4.094	1.622	6.568	261.007	485000	187000
0	0	Early	2.565	0.122	0.463	76.461	516000	167000
0	0	Early	2.485	4.506	16.499	481.03	521000	148000
1	1	Late	3.829	0.324	1.431	150.941	501000	144000
0	0	Early	2.197	9.142	27.51	533.807	596000	275000
0	0	Early	2.079	7.228	23.81	556.657	501000	207000
1	0	Early	2.708	1.141	4.318	111.457	613000	167000
1	0	Late	3.689	9.639	35.647	746.187	451000	224000
1	1	Early	3.714	6.173	23.047	472.916	506000	345000
1	0	Early	3.219	0.028	0.077	31.131	413000	280000
0	0	Late	2.303	0.7	2.971	164.706	415000	115000
1	0	Late	2.89	4.249	17.039	298.051	499000	126000
1	0	Early	2.89	0.218	0.751	67.581	483000	238000
0	0	Early	2.398	0.982	2.616	74.978	615000	246000
1	0	Late	3.296	0.017	0.084	15.39	539000	188000
0	0	Late	3.526	5.129	16.215	390.368	619000	239000
0	0	Late	3.434	0.495	1.187	114.893	369000	610000
0	0	Early	2.398	3.808	5.755	466.396	330000	281000
1	0	Late	3.912	1.311	4.737	176.147	468000	106000
1	0	Late	3.989	1.996	6.532	206.507	544000	195000
1	1	Early	3.178	1.573	3.912	239.417	397000	228000
1	0	Early	2.303	0.697	2.434	161.801	516000	150000
1	0	Early	3.178	4.177	13.698	375.563	583000	161000
0	0	Late	3.367	0.477	0.933	85.287	411000	278000
1	0	Early	2.708	5.199	18.135	337.677	582000	215000
0	1	Early	1.792	1.969	4.723	480.525	374000	229000
0	0	Early	2.079	2.707	7.363	418.236	332000	125000
1	0	Early	2.303	9.86	20.103	1017.772	361000	227000
0	0	Early	2.485	2.55	8.504	314.063	297000	103000
0	1	Early	2.485	7.719	14.353	539.464	443000	438000
1	0	Early	2.079	1.508	3.259	306.159	316000	234000

0	0	Late	3.178	0.252	0.431	103.567	294000	181000
1	0	Late	3.332	5.112	10.691	391.669	336000	124000
1	0	Late	2.639	2.55	8.434	282.336	502000	131000
0	0	Early	2.197	6.24	18.272	424.089	498000	364000
1	0	Early	2.708	1.628	4.011	251.341	209000	65000
1	0	Early	3.091	0.711	1.771	93.806	620000	310000
1	1	Early	2.565	4.749	9.059	476.422	398000	255000
1	0	Early	2.197	3.385	8.417	396.876	413000	226000
1	0	Early	2.398	3.112	9.288	398.329	436000	210000
1	0	Late	3.466	0.601	1.03	102.025	416000	501000
0	0	Late	3.401	1.726	2.658	195.302	418000	498000
0	1	Early	3.091	3.898	10.515	662.531	376000	222000
1	0	Early	2.773	3.99	15.983	363.482	458000	107000
1	0	Late	3.178	2.732	9.534	274.79	517000	207000
0	0	Early	3.367	0.142	0.441	62.91	509000	175000
0	0	Late	4.127	9.812	30.069	736.486	502000	215000
0	0	Late	3.611	0.027	0.174	20.755	523000	186000
0	0	Early	2.485	8.608	28.854	793.768	423000	187000
1	0	Early	2.639	5.037	16.787	361.649	568000	162000
1	0	Early	2.485	1.357	3.122	291.582	393000	334000
0	0	Late	3.045	1.624	5.585	284.114	538000	128000
1	0	Late	2.773	0.097	0.224	64.146	402000	393000
1	0	Early	2.303	7.005	23.274	484.924	527000	217000
1	0	Early	2.485	2.262	5.624	379.211	371000	224000
1	0	Early	2.565	4.405	9.563	492.252	328000	138000
0	0	Early	2.708	0.019	0.046	24.463	257000	354000
1	0	Early	3.401	0.078	1.332	82.556	512000	175000
0	0	Late	3.091	3.779	13.47	311.793	524000	205000
1	0	Early	2.398	2.14	7.921	269.373	529000	153000
1	0	Early	2.485	9.646	16.812	586.455	451000	392000
1	0	Early	2.944	0.975	1.817	217.485	367000	177000
0	1	Early	2.773	0.112	0.29	27.09	529000	180000
0	0	Early	2.197	3.293	13.84	461.96	461000	153000
1	0	Early	2.303	0.336	0.701	105.013	398000	227000
0	0	Late	3.714	1.776	6.987	317.666	506000	158000
0	1	Late	3.611	0.125	0.716	98.648	461000	85000
0	0	Late	3.784	0.114	0.482	88.41	516000	168000
0	0	Late	3.807	0.063	0.313	64.78	521000	160000
0	0	Early	3.219	1.638	5.06	176.44	552000	154000
1	0	Early	2.565	6.634	13.339	525.281	303000	141000
0	0	Late	3.638	9.633	30.666	630.536	495000	353000
1	0	Early	3.296	0.142	0.41	65.707	491000	169000
1	0	Late	3.434	8.499	36.088	613.704	465000	130000
1	0	Late	2.197	0.185	0.963	284.382	227000	643000
1	0	Early	2.079	2.846	12.584	406.767	416000	126000
1	1	Late	3.584	6.534	12.639	799.488	401000	242000

0	0	Late	3.258	0.69	3.738	218.073	467000	174000
0	1	Late	2.398	6.173	23.047	472.916	506000	345000
0	0	Early	1.946	10.232	33.203	914.928	520000	225000
0	1	Late	3.045	4.506	16.499	481.03	521000	148000
1	0	Late	2.773	11.554	37.372	596.413	557000	235000
0	0	Early	2.079	0.417	0.809	196.204	334000	221000
1	0	Late	3.178	2.846	12.584	406.767	416000	126000
1	0	Late	2.89	7.005	23.274	484.924	527000	217000
0	0	Early	2.485	0.816	2.996	234.796	334000	127000
1	0	Late	3.045	0.06	0.185	28.725	489000	158000
1	0	Late	3.401	7.625	30.626	768.199	442000	177000
1	0	Early	3.135	0.044	0.175	82.114	257000	561000
0	0	Late	2.89	13.411	21.891	875.764	471000	432000
1	0	Late	3.258	2.873	5.925	431.662	373000	247000
1	0	Early	2.485	8.084	22.644	509.711	589000	227000
0	1	Late	3.091	0.076	0.256	42.563	542000	178000
1	0	Late	3.497	7.764	17.233	471.595	470000	343000
0	0	Early	2.485	1.416	2.596	123.463	504000	429000
0	0	Early	3.178	0.986	2.313	132.018	325000	390000
1	0	Early	2.303	5.553	19.725	517.252	428000	209000
0	0	Late	3.091	6.358	14.242	409.375	470000	375000
1	0	Late	3.611	6.522	19.168	705.045	566000	149000
0	1	Early	2.708	6.218	14.276	1107.356	377000	829000
0	0	Early	2.944	1.575	5.42	278.007	538000	128000
0	0	Late	3.045	0.793	1.267	111.016	170000	45000
0	0	Early	2.485	0.2	0.76	84.326	490000	143000
0	0	Late	3.689	0.052	0.151	17.489	535000	176000
1	1	Late	2.833	2.289	7.092	287.196	234000	64000
0	0	Late	3.85	0.153	0.361	115.02	382000	556000
1	0	Early	2.565	0.074	0.278	46.042	524000	169000
1	0	Late	2.944	3.767	10.156	326.735	576000	289000
1	0	Early	1.946	9.948	37.249	662.548	469000	229000
1	0	Late	3.434	0.125	0.305	83.212	401000	446000
0	1	Early	2.773	0.491	1.402	93.444	212000	51000
0	0	Late	2.398	2.608	9.21	311.974	369000	112000
0	0	Early	3.135	0.02	0.051	18.012	400000	427000
1	0	Late	3.807	11.022	42.831	1070.149	456000	187000
1	0	Early	1.946	8.203	29.722	603.658	453000	221000
1	0	Late	3.555	0.044	0.175	82.114	257000	561000
0	0	Late	2.639	1.323	2.723	285.179	337000	447000
1	0	Late	3.526	8.141	16.812	578.249	435000	295000
0	0	Late	2.079	0.335	0.791	155.184	320000	474000
1	0	Late	3.178	4.539	19.229	486.618	387000	115000
1	1	Late	3.555	1.573	3.912	239.417	397000	228000
1	0	Late	3.178	3.808	5.755	466.396	330000	281000
0	0	Late	3.526	1.046	3.545	185.702	418000	188000

1	1	Late	2.944	0.742	3.518	197.481	341000	107000
1	0	Late	3.555	0.489	1.603	141.795	349000	106000
0	0	Late	2.639	3.817	11.027	338.473	607000	155000
1	0	Late	4.159	0.627	2.167	109.477	528000	196000
1	0	Late	3.466	0.171	0.4	105.852	402000	350000
0	1	Late	2.944	0.586	2.419	137.625	608000	160000
0	0	Late	3.135	1.638	6.421	255.804	479000	169000
1	0	Late	3.219	9.614	29.444	731.517	405000	220000
0	0	Late	3.332	1.684	3.603	222.863	374000	163000
1	0	Late	3.258	2.033	7.965	409.501	394000	173000
1	1	Late	3.434	1.741	8.045	317.149	475000	182000
0	1	Late	3.258	6.218	14.276	1107.356	377000	829000
0	1	Late	3.664	0.245	0.546	66.04	352000	206000
0	0	Late	3.784	7.232	24.463	669.209	395000	193000
1	0	Late	3.497	3.809	12.619	411.768	401000	162000
1	0	Late	3.135	2.29	3.163	208.371	458000	379000
0	0	Late	3.497	0.135	0.285	78.845	352000	492000
0	0	Late	3.97	2.377	7.645	431.268	457000	106000
0	0	Late	3.135	0.052	0.168	31.32	489000	159000
0	0	Late	3.784	5.661	19.91	975.323	347000	735000
1	0	Late	3.738	0.177	0.376	125.686	378000	444000
1	0	Late	3.892	0.341	1.154	97.2	550000	173000
1	0	Late	2.303	0.035	0.203	85.821	185000	731000
1	1	Late	4.078	0.283	0.554	166.832	260000	873000
1	0	Late	3.091	0.975	1.817	217.485	367000	177000
0	0	Late	3.219	0.819	1.49	230.444	355000	180000
0	0	Late	3.332	0.068	0.234	37.392	534000	173000
0	0	Late	3.401	12.112	35.209	614.08	601000	267000
1	0	Late	3.296	0.024	0.097	17.185	525000	170000
0	0	Late	3.526	1.55	4.454	289.472	512000	164000
1	0	Late	2.708	1.545	2.773	129.406	454000	319000
0	0	Late	3.091	0.194	0.553	63.931	496000	165000
0	1	Late	3.178	6.472	11.323	395.415	462000	337000
0	0	Late	3.178	12.774	26.526	675.014	475000	376000
1	0	Late	3.555	5.453	20.545	584.562	499000	106000
0	1	Late	3.664	0.262	0.315	135.117	268000	258000
0	0	Late	3.367	1.624	5.585	284.114	538000	128000
0	0	Late	2.833	9.948	37.249	662.548	469000	229000
0	0	Late	3.045	6.032	14.153	1383.854	302000	860000
0	0	Early	2.996	3.231	10.361	316.15	508000	204000
0	0	Late	3.219	2.828	5.926	244.964	401000	571000
1	0	Late	4.007	0.847	4.45	264.4	504000	157000
1	0	Late	3.829	2.215	7.398	316.689	493000	100000
1	0	Late	3.296	3.273	5.4	528.368	329000	250000
0	0	Late	4.477	2.196	7.801	219.497	621000	245000
1	0	Late	3.584	0.574	2.21	259.92	349000	844000

0	0	Late	2.944	10.398	30.345	660.374	536000	360000
1	0	Late	2.773	2.337	6.272	210.203	273000	44000
0	0	Late	4.263	6.959	17.599	667.68	418000	236000
1	0	Late	3.611	1.416	2.596	123.463	504000	429000
1	0	Late	2.639	11.874	34.661	534.394	597000	260000
1	0	Late	2.944	6.729	23.737	972.993	343000	738000
1	0	Late	3.367	0.608	1.822	110.147	571000	158000
1	0	Late	2.944	7.181	12.479	449.209	462000	482000
1	0	Late	3.664	0.094	0.159	32.459	439000	385000
1	1	Late	3.258	0.078	1.332	82.556	512000	175000
1	0	Late	3.638	6.667	21.98	544.896	524000	234000
0	1	Late	3.219	10.717	27.136	533.608	598000	268000
1	0	Late	3.497	8.327	17.985	789.849	393000	246000
1	1	Late	3.219	0.02	0.042	18.371	290000	193000
0	0	Late	3.526	3.967	12.449	511.916	401000	195000
1	0	Late	3.434	4.249	17.039	298.051	499000	126000
1	0	Late	3.332	2.738	8.905	321.287	550000	163000
1	0	Late	3.466	7.189	18.167	400.122	618000	278000
0	0	Late	3.638	0.018	0.045	14.772	432000	386000
0	0	Late	2.708	0.051	0.255	51.842	514000	171000
1	1	Late	3.526	1.141	4.318	111.457	613000	167000
0	0	Late	4.143	10.755	18.521	782.873	462000	439000
1	0	Late	3.401	0.545	1.065	50.615	426000	568000
1	0	Late	3.526	0.281	0.568	210.642	227000	332000
0	0	Late	3.401	7.921	23.907	390.102	587000	327000
1	0	Late	3.178	3.294	5.818	243.412	451000	480000
0	0	Late	3.091	13.066	21.114	862.54	458000	428000
0	0	Late	3.258	4.4	15.059	502.944	400000	158000
1	0	Late	3.367	4.99	15.448	743.852	303000	686000
0	0	Late	2.639	3.898	10.515	662.531	376000	222000
1	0	Early	3.401	0.222	0.346	113.538	211000	208000
1	0	Late	3.871	0.526	2.489	162.349	534000	125000
0	0	Late	3.401	0.369	0.654	122.762	356000	175000
0	0	Late	3.401	0.13	0.428	48.811	519000	170000
1	0	Late	2.485	0.336	0.613	85.673	378000	395000
1	0	Late	4.111	2.457	7.206	259.791	614000	133000
0	0	Late	3.296	1.729	5.209	277.751	466000	244000
1	0	Late	3.434	0.559	1.824	208.97	372000	168000
0	0	Late	3.829	6.157	20.784	491.603	530000	213000
1	0	Late	3.258	0.337	1.429	260.426	400000	186000
1	0	Late	3.401	2.29	3.163	208.371	458000	379000
1	1	Late	3.584	0.06	0.177	30.16	535000	191000
1	1	Late	3.784	8.559	28.895	858.016	410000	157000
1	0	Late	2.89	4.197	13.303	572.622	393000	176000
0	0	Late	2.944	8.322	20.206	367.63	616000	303000
0	0	Late	3.258	0.087	0.217	75.289	395000	549000

1	0	Late	3.219	0.398	0.838	94.736	411000	553000
1	0	Late	3.296	3.701	12.658	471.584	399000	158000
1	0	Late	3.178	0.268	0.416	172.746	256000	257000
0	0	Late	3.932	2.297	6.24	295.612	329000	378000
1	0	Late	2.944	13.003	39.14	1055.564	528000	322000
1	0	Late	3.091	0.658	2.128	239.738	363000	110000
0	0	Late	2.944	3.914	10.791	426.903	382000	132000
0	0	Late	3.332	0.199	0.258	70.244	292000	181000
0	0	Late	3.401	9.86	20.103	1017.772	361000	227000
0	0	Late	3.219	0.22	0.679	65.959	252000	70000
1	1	Late	3.611	2.086	5.637	254.676	251000	137000
0	0	Late	3.611	10.061	31.397	1034.031	517000	241000
0	0	Late	3.045	0.023	0.058	20.805	395000	430000
0	0	Late	3.784	10.232	33.203	914.928	520000	225000
0	0	Late	3.296	2.712	7.941	282.714	475000	257000
0	0	Late	3.871	0.25	0.637	108.183	295000	139000
1	1	Late	3.989	0.012	0.059	11.454	527000	172000
0	0	Late	2.833	2.089	3.429	253.446	433000	316000
1	0	Late	1.609	2.616	5.393	247.052	436000	290000
0	0	Late	3.045	6.879	15.152	459.636	422000	615000
1	0	Late	3.951	0.043	0.11	39.579	387000	422000
1	0	Late	3.611	7.046	23.583	588.85	447000	226000
1	0	Late	2.944	0.843	3.141	249.635	334000	127000
0	0	Late	3.689	0.119	0.415	32.338	618000	243000
0	0	Late	2.89	0.585	1.462	130.56	360000	206000
1	0	Late	3.829	5.802	10.114	402.221	433000	543000
1	0	Late	3.638	0.579	1.866	219.561	387000	375000
1	0	Late	2.944	4.046	12.893	465.822	547000	155000
1	0	Late	3.871	0.179	0.374	187.897	243000	374000
1	0	Late	3.466	0.169	0.493	60.551	495000	157000
1	0	Late	3.714	0.528	1.946	135.288	522000	105000
1	0	Late	3.664	0.159	0.361	38.218	333000	386000
0	0	Late	3.555	0.14	0.274	78.007	278000	376000
0	0	Late	3.951	3.673	14.658	574.863	449000	192000
1	0	Late	3.85	1.039	2.967	101.898	547000	167000
1	0	Late	3.664	0.186	0.417	127.774	401000	347000
0	1	Late	4.078	6.104	29.319	476.067	394000	111000
1	1	Late	3.555	3.678	8.859	612.528	344000	324000
0	0	Late	3.332	3.093	10.547	406.891	421000	221000
0	0	Late	3.738	1.882	4.887	274.465	355000	634000
1	0	Late	3.466	3.061	6.962	330.001	405000	262000
1	0	Late	3.466	3.926	12.65	560.74	489000	148000
0	1	Late	3.466	0.748	2.08	139.933	211000	52000
0	0	Late	4.369	0.008	0.03	4.333	534000	182000
0	0	Late	3.091	0.208	0.777	81.213	490000	143000
1	0	Late	2.996	0.753	2.05	190.788	507000	167000

0	0	Late	3.178	0.349	0.964	100.122	247000	74000
0	0	Late	3.664	0.745	2.861	140.23	481000	152000
1	1	Late	3.497	1.415	4.974	263.331	483000	167000
1	0	Late	3.689	6.405	11.317	390.738	432000	411000
0	0	Late	3.638	0.105	0.275	85.873	379000	586000
1	0	Late	3.638	0.1	0.374	57.817	521000	163000
1	0	Late	4.205	0.051	0.089	19.269	509000	432000
1	0	Late	3.638	2.099	7.753	413.26	520000	140000
1	0	Late	4.025	3.385	8.417	396.876	413000	226000
1	0	Late	3.555	5.272	16.478	497.037	523000	223000
1	1	Late	2.944	2.644	6.399	527.934	375000	821000
0	1	Late	3.829	0.553	0.993	133.344	333000	160000
1	0	Late	3.434	1.348	2.772	161.207	159000	28000
1	0	Late	3.989	4.403	7.605	326.509	456000	319000
0	0	Late	2.833	0.67	1.389	183.289	338000	161000
0	0	Late	3.784	1	2.323	239.948	345000	550000
0	0	Late	3.434	1.369	4.877	159.557	469000	111000
0	0	Late	4.007	6.933	20.477	657.312	550000	165000
1	0	Late	3.97	11.683	24.231	641.826	474000	371000
1	0	Late	3.689	1.441	4.819	320.365	392000	163000
1	0	Late	3.829	2.733	11.938	343.035	464000	175000
0	0	Late	4.443	4.368	14.896	563.599	489000	148000
1	0	Late	3.526	0.291	1.211	164.648	555000	129000
0	0	Late	3.178	0.043	0.11	39.051	393000	425000
1	0	Late	3.807	1.485	3.084	220.674	332000	158000
1	1	Late	3.951	3.99	15.983	363.482	458000	107000
0	0	Late	3.584	5.051	10.606	504.562	469000	383000
0	0	Late	3.555	2.099	6.551	345.352	394000	188000
1	0	Late	2.639	0.071	0.164	68.395	313000	188000
1	0	Late	3.296	0.489	1.861	235.825	352000	809000
1	0	Late	3.829	7.611	17.227	452.099	514000	420000
1	0	Late	3.497	0.78	1.863	110.97	292000	55000
0	0	Late	3.555	8.587	27.287	737.335	403000	163000
1	0	Late	3.258	10.093	31.093	568.168	594000	199000
1	0	Late	3.714	0.171	0.683	66.266	490000	142000
1	0	Late	4.127	7.44	22.912	487.497	527000	216000
1	0	Late	3.932	0.224	0.696	70.171	541000	134000
1	0	Late	4.369	0.06	0.244	46.838	544000	171000
1	0	Late	3.091	6.713	29.61	576.895	466000	138000
1	0	Late	3.829	0.214	0.555	51.696	537000	184000
0	1	Late	3.871	0.185	0.963	284.382	227000	643000
0	0	Late	4.143	0.751	1.647	226.25	335000	158000
0	0	Late	3.332	1.732	5.437	499.01	368000	207000
1	1	Late	3.638	0.132	0.285	95.293	345000	523000
0	0	Late	3.807	6.086	21.646	553.535	428000	209000
0	0	Late	3.332	0.703	1.975	107.354	225000	67000

0	0	Late	3.555	0.125	0.305	83.212	401000	446000
1	1	Late	3.296	0.338	0.772	204.882	235000	377000
1	0	Late	3.466	2.632	11.678	344.764	371000	91000
1	0	Late	3.466	0.283	0.635	72.627	385000	254000
1	0	Late	3.989	0.13	0.404	39.643	520000	169000
0	0	Late	2.944	0.049	0.1	33.98	395000	425000
1	0	Late	4.477	8.712	26.409	590.609	594000	273000
0	0	Late	4.007	5.659	22.124	446.837	499000	203000
0	0	Late	3.401	2.884	8.966	541.426	396000	164000
1	0	Late	3.689	0.42	1.983	107.521	470000	184000
1	1	Late	3.219	0.015	0.038	13.318	400000	427000
1	0	Late	3.611	5.991	13.912	494.581	590000	292000
0	0	Late	3.714	0.651	2.018	188.559	518000	130000
0	0	Late	3.091	14.043	44.662	748.956	563000	209000
0	0	Late	3.892	0.191	0.642	93.671	515000	167000
1	0	Late	3.367	8.797	27.116	512.977	510000	300000
1	0	Late	3.989	0.03	0.147	29.166	520000	177000
1	1	Late	2.485	0.079	0.126	29.162	427000	435000
1	0	Late	2.639	0.374	1.012	165.302	355000	107000

Table S6. This table shows averaged bipartite food web metrics (based on random picks of 5 hives) from honey collected early (≤ June) or late (≥ August) season within the following arable percentage cover band classes of 0-10 %, 10-20 %, 20-30 %, 30-40 %, 40-50 %, 50-60 %, 60-70% and 70-90%. The number of hives in each of these bands is given in the table. The bipartite food webs were used to derive weighted connectance (realised proportion of possible links between hives and plants), weighted NODF nestedness (the tendency for hives to forage on subsets of plants utilised by better-connected hives, where larger values indicate increased nestedness), niche overlap (mean similarity of interaction patterns for hives with plants) and generality (mean effective number of plants foraged upon per hive).

	**Total number of hives**	**Arable area (Band mid-point)**	**Season**	**weighted connectance**	**weighted NODF**	**Niche.overlap.HL**	**Generality.HL**
Early_0to10	38	5	Early	0.064	22.51	0.434	5.592
Early_10to20	12	15	Early	0.081	23.905	0.534	4.094
Early_20to30	18	25	Early	0.092	25.236	0.58	4.356
Early_30to40	9	35	Early	0.088	25.359	0.798	4.969
Early_40to50	11	45	Early	0.14	38.954	0.89	3.546
Early_50to60	12	55	Early	0.1	31.021	0.605	4.526
Early_60to70	9	65	Early	0.125	33.391	0.824	3.413
Early_70to90	10	85	Early	0.167	44.151	0.983	3.151
Late_0to10	175	5	Late	0.041	19.327	0.327	6.799
Late_10to20	47	15	Late	0.044	19.114	0.413	7.343
Late_20to30	47	25	Late	0.043	20.206	0.452	6.804
Late_30to40	33	35	Late	0.048	22.87	0.357	7.262
Late_40to50	26	45	Late	0.047	20.59	0.406	8.006
Late_50to60	26	55	Late	0.048	21.423	0.403	8.063
Late_60to70	24	65	Late	0.048	22.334	0.284	6.073
Late_70to90	30	85	Late	0.048	20.943	0.272	7.52

Table S7. Due to cost restrictions it was not possible to assess all 527 honey samples for agrochemical residues. Instead analyses undertaken in the paper assessed potential exposure to pesticides based on the UKCEH Land Cover plus: Pesticides 2012-2017 map [1]. However, 100 randomly selected honey samples from 2019 were tested for pesticides. Residues were assessed using the QuEChERS (Quick, Easy, Cheap, Effective and Safe) method [2]. This analysis was undertaken using liquid chromatography coupled to a triple quadrupole 'Quantum Ultra TSQ' mass spectrometer (Waters ACQUITY UPLC system coupled with a Waters XEVO TQ-XS Tandem Mass Spectrometer; UK) interfaced with a Unispray source operated using Masslynx software (version 4.2). Quantification of pesticides was based on their respective response factor compared to the internal standards and the chromatographic peaks assessed using the Waters Targetlynx software (part of the Masslynx suite). We used logistic regression to assess the probability of these pesticides being detected in honey in response to their predicted application rate within a 2 km radius of hives as determined from the UKCEH Land Cover plus: Pesticides 2012-2017 map. This was restricted to compounds with a minimum 30 % detection rate across the 100 samples to ensure there was sufficient data density for a robust analysis. The majority of these frequently encountered agrochemicals were fungicides including the azole fungicides cyproconazole and tebuconazole. In each case the full model (probability of detecting active ingredient = predicted application rate from UKCEH Land Cover plus: Pesticides 2012-2017 map) was compared to a null intercept only model based on Akaike information criterion (AIC). The model with the lowest value was considered the best fit to the data, with models with greater than 2 AIC point difference being robust [3]. Appropriate tests of model assumptions based on residuals were undertaken. Of these compounds detection in honey was positively correlated with the application rate surrounding hives predicted by the UKCEH Land Cover plus: Pesticides 2012-2017 map. The exception was the fungicide boscalid which was almost ubiquitous in honey samples, being present in 95 % of those tested. Note, Boscalid has low toxicity to bees and to our knowledge there is no evidence of it being associated with synergistic interactions with other pesticides. The below table shows the AIC scores comparing the null to full models as described above, with individual active ingredient plots of the probability of detection against the predicted application rates based on the UKCEH Land Cover plus: Pesticides 2012-2017 map below this.

Active ingredient	Correlated	Null-model AIC	Full application rate model AIC
Azoxystrobin	Yes (+ve)	140.2	133.4
Boscalid	No	67.01	67.5
Cyproconazole	Yes (+ve)	93.17	89.6
Propamocarb	Yes (+ve)	138.1	133.4
Tebuconazole	Yes (+ve)	139.6	137.6
Thiacloprid	Yes (+ve)	140.4	128.1

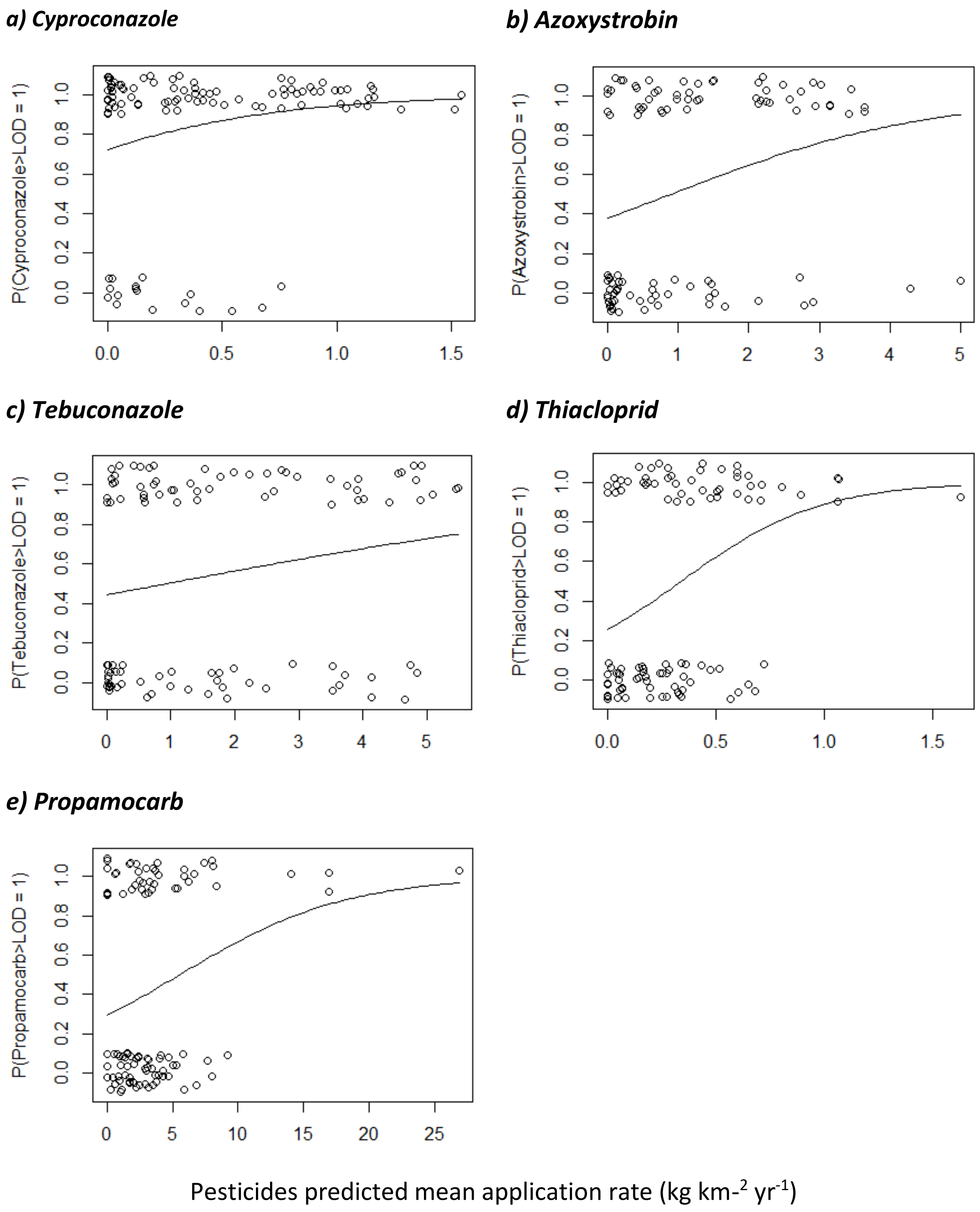

References

1 Jarvis, S. G. *et al.* CEH Land Cover plus: Pesticides 2012-2017 (England, Scotland and Wales). *NERC Environmental Information Data Centre.* , doi:https://doi.org/10.5285/99a2d3a8-1c7d-421e-ac9f-87a2c37bda62 (2020).

2 Li, J. *et al.* Enantioselective determination of triazole fungicide simeconazole in vegetables, fruits, and cereals using modified QuEChERS (quick, easy, cheap, effective, rugged and safe) coupled to gas chromatography/tandem mass spectrometry. *Analytica Chimica Acta* **702**, 127-135, doi:10.1016/j.aca.2011.06.034 (2011).

3 Burnham, K. P. & Anderson, D. R. *Model selection and multimodel inference: a practice information-theoretic approach.*, (Springer, 1998).
